# Palatability and Stability Studies to Optimize a Carvedilol Oral Liquid Formulation for Pediatric Use

**DOI:** 10.3390/pharmaceutics16010030

**Published:** 2023-12-25

**Authors:** Blanca Chiclana-Rodríguez, Encarnacion Garcia-Montoya, Miquel Romero-Obon, Khadija Rouaz-El-Hajoui, Anna Nardi-Ricart, Marc Suñé-Pou, Josep M. Suñé-Negre, Pilar Pérez-Lozano

**Affiliations:** 1 Department of Pharmacy and Pharmaceutical Technology and Physical Chemistry, Faculty of Pharmacy and Food Sciences, University of Barcelona, Av. Joan XXIII, 27-31, 08028 Barcelona, Spain; blachirod@gmail.com (B.C.-R.); miquel.romero@ub.edu (M.R.-O.); khadijarouaz@ub.edu (K.R.-E.-H.); annanardi@ub.edu (A.N.-R.); marcsune@ub.edu (M.S.-P.); jmsune@ub.edu (J.M.S.-N.); perezlo@ub.edu (P.P.-L.); 2 Pharmacotherapy, Pharmacogenetics and Pharmaceutical Technology Research Group, Bellvitge Biomedical Research Institute (IDIBELL), Av. Gran Via de l’Hospitalet, 199-203, 08090 Barcelona, Spain

**Keywords:** carvedilol, pediatrics, palatability, bitterness, taste assessment, human taste panel, stability, flavorings

## Abstract

Carvedilol (CARV) is a blocker of α- and β- adrenergic receptors, used as an “off-label” treatment for cardiovascular diseases in pediatrics. Currently, there is no marketed pediatric-appropriate CARV liquid formulation, so its development is necessary. Palatability (appreciation of smell, taste, and aftertaste) is a key aspect to be considered during the development of pediatric formulations since only formulations with good palatability also have adequate acceptability in this population. Consequently, the aim of this research was to assess the palatability and acceptability of different CARV formulations using an in vivo taste assessment (ID Number PR103/22) in order to select the highest palatability-rated CARV formulation. The preparation of CARV formulations was based on a reference 1 mg/mL CARV solution, which contains malic acid as a solubilizing agent. Subsequently, sucralose and flavoring agents were added and mixed until complete dissolution to the corresponding formulations. Adult volunteers participated in this study and evaluated the taste and odor of various CARV formulations through a questionnaire and a sensory test. The mean palatability score, measured on a 10-point scale, increased from 1.60 for the unflavored control to 7.65 for the highest-rated flavored formulation. Moreover, the bitterness of the optimized CARV formulation was reduced from 66.67% to 17.86%, and the taste pleasantness was increased from 25/100 to 73/100. This optimized CARV formulation contains a sweetening agent, sucralose, in addition to two flavoring agents at appropriate concentrations for pediatrics. Furthermore, the physicochemical and microbiological stability of the optimized CARV formulation were evaluated for 6 months at 25, 30, and 40 °C, in addition to in-use stability for 15 days at 25 °C, whose results were confirmed. Thus, we successfully developed a palatable CARV liquid solution that contains excipients appropriate for pediatrics and is stable under the studied conditions.

## 1. Introduction

Oral liquid formulations are often deemed optimal for pediatric application, a viewpoint also supported by the European Medicines Agency (EMA). This preference primarily stems from their flexibility in dosing and adaptability, allowing drug dosage to be tailored according to the age and weight of each pediatric patient [1]. Designing a pediatric oral formulation is challenging with respect to several issues, such as drug solubility and the selection of excipients, which greatly influence palatability. The palatability of a pharmaceutical formulation refers to its level of pleasantness or acceptability concerning taste, smell, and the overall sensory experience upon administration to patients. Oral pharmaceutical formulations are often unpalatable. Hence, it is often cited as a major cause of non-adherence, especially for geriatric and pediatric populations, who may have swallowing difficulties. An unpalatable formulation can heavily reduce the young patient’s compliance and, consequently, the effectiveness of the therapy [2].

Various taste-masking strategies can be employed for this purpose, and it is important to account for children’s preferences. However, it is crucial to note that certain excipients might elicit adverse reactions in children, particularly when treating infants and neonates. The EMA [1,3] has issued guideline/reflection papers for the pediatric population, while the FDA (Food and Drug Administration) has created a set of guidance documents centered around pediatric patient-focused drug development. Additionally, taste-masking assessments were listed in the contents of the investigation of pediatric drug product development plan measures specified in the pediatric regulations [4,5].

Numerous taste-masking methods exist, yet none universally meet the needs of all drug types, posing a challenge in selecting suitable technology for various Active Pharmaceutical Ingredients (APIs). Taste masking approaches have been primarily categorized based on the mechanism of taste transmission. One approach involves blocking taste transmission pathways, employing methods such as flavoring agents and bitter inhibitors. Generally, the first choice is to use flavoring agents to mask the taste. After that, if the taste is still unacceptable, we should research other methods or a combination of them. The incorporation of flavors serves to mitigate the bitter taste of medicine by competing with the drugs to stimulate taste receptor cells. For example, sweeteners are found to be more effective in masking bitterness compared to acid agents [6,7]. Flavors are categorized as a type of excipient that inherently possesses a pleasant taste and odor. These flavoring agents are commonly classified into sweeteners, acidic flavors, and aromatic agents based on their taste. From the pharmaceutical industry’s point of view, adding flavors is a very attractive method because of its inherently enjoyable taste. There are numerous sweeteners in practical application, such as sucrose, lactose, aspartame, sucralose, mannitol, and saccharin sodium. Meanwhile, the acceptable daily intake (ADI) of sweeteners is also worthy of attention, especially for children. Other frequently used taste-masking techniques for oral formulations include lipophilic vehicles, cyclodextrin complexation, ion exchange resin, liposomes, microcapsules, and nanoemulsions [7,8].

CARV is a non-selective blocker of α- and β-adrenergic receptors used as an ‘off-label’ treatment for clinically treating cardiovascular diseases such as hypertension and congestive heart failure in the pediatric population. Additionally, EMA has included carvedilol on its list of pediatric and therapeutic requirements for cardiology. However, CARV has only been commercialized in an oral solid dosage form as a tablet; hence, the development of a pediatric-appropriate pharmaceutical formulation for CARV is necessary [9]. We successfully developed a stable aqueous CARV liquid formulation suitable for pediatrics [10]. However, optimization of this formulation is the next step, which includes taste assessment and a stability study in order to make CARV formulation more palatable. CARV dissolved in water exhibits an unpleasant bitter taste, which is challenging to mask, making the formulations less palatable. Therefore, palatability was selected as the criterion of choice for a new palatable age-adapted formulation.

CARV tablets are often manipulated prior to use in hospitals with the aim of improving patient compliance and adherence to prepare a CARV liquid suspension [10]. The most common excipient used to prepare a suspension with triturated CARV tablets is a mixture of Ora-Sweet–Ora-Plus (1:1) or Ora-Blend. Some components of this mixture, such as sorbitol, sucrose, and saccharin, are not recommended in pediatrics [11,12]. Ora-sweet gave a sweet citrus-berry flavor to the formulation. Another example is SyrSpend SF PH4 (Fagron), a ready-to-use, all-in-one suspending and sweetening oral liquid vehicle containing cherry flavoring [13].

Operto et al. [14] developed two CARV liquid formulations for administration to pediatric. However, to improve the palatability of these formulations, a small volume of the formulation may be diluted in milk prior to administration. Combining a formulation with food or drinks, such as milk, to mask its unpleasant taste may be considered if it is proven that further improvement is not achievable. However, this can influence the pharmacokinetic behavior of the API [3].

Furthermore, the development of CARV mini tablets could avoid the bitter, unpleasant taste of the API. Khan et al. [15] developed a CARV orally disintegrating mini tablet (ODMT) appropriate for pediatrics, whose doses were 0.5 mg and 2 mg, compared to 3.125 mg as the lowest strength of CARV-marketed tablets. However, oral solutions remain the preferred choice for pediatrics, especially in neonates or children up to 4 years old, as well as for elderly patients who have swallowing difficulties [3].

The taste of a pharmaceutical oral liquid formulation can be quantitatively evaluated using a taste sensor as an electronic tongue (e-tongue) or qualitatively by taste panels. The e-tongue functions were akin to human gustatory sensation, capable of detecting tastes by changes in the electric charge density on the sensor’s membrane surface when exposed to taste substances. The electronic tongue provides an output indicating the taste quality and intensity of the tested formulations compared to predetermined references. Some researchers used an e-tongue for the development of a palatable pediatric formulation [1,16,17].

However, human testing using a sensory questionnaire is acknowledged as the best method to assess the taste of a pharmaceutical formulation. Sensory results of taste assessment in human volunteers may be closer to the real, compared to sensory results using an e-tongue [7]. It is evident that children as a target population are regarded as the most suitable panel for taste assessment of pediatric formulations because children experience different taste sensations than adults [18]. Nevertheless, authorities recommend conducting palatability assessments involving children whenever feasible. When such assessments with children are not possible, taste screening by adult panels can serve as an alternative method, with the results examined for their applicability to children [19]. At this stage of development, we deemed an initial screening by an adult testing panel to be acceptable. Regarding the palatability evaluation by healthy volunteers, each healthy adult volunteer was asked to evaluate their taste and odor perception using a questionnaire rating scale.

Considering these findings, the goal of this work was the development of an oral palatable solution of CARV for pediatric use, exploiting taste masking in association with different flavoring agents and a sweetener. Different combinations of flavorings and a sweetening agent were selected to prepare CARV solutions and were subject to a human taste assessment to evaluate their actual ability to mask the unpleasant taste of the CARV in comparison with the reference CARV solution [10].

The aim of this research was to select the CARV solution with the highest palatability score and least bitterness level for healthy human volunteers. The highest palatability-rated CARV formulation for the participants was included in a stability study for 6 months and evaluated in terms of chemical, physical, and microbiological stability under 25, 30, and 40 °C. Additionally, a 15-day in-use stability study of this formulation was conducted at 25 °C.

## 2. Materials and Methods

### 2.1. Materials

European Pharmacopoeia grade (Ph. Eur) carvedilol was kindly donated by MOEHS (Barcelona, Spain). Ph. Eur. grade malic acid-DL and sucralose were purchased from FAGRON IBERICA (Barcelona, Spain).

Flavoring agent samples were donated by the Kerry Group (Kerry Iberia Taste & Nutrition; Barcelona, Spain) and GIVAUDAN IBERICA (Barcelona, Spain). Characteristics of flavoring agents are presented in Table 1. The water used for analysis was MilliQ grade. All solvents used were analytical grade.

Sucralose was chosen due to its safety profile and high sweetness. It possesses a sweetening potency approximately 300 to 1000 times greater than sucrose, lacks an aftertaste, is non-cariogenic, devoid of nutritional value, does not contribute to dental cavities, and does not elicit a glycemic response [20].

The Acceptable Daily Intake (ADI) for sucralose in the EU is set at 15 mg/kg/day, as recommended by the Scientific Committee on Food of the European Commission (SCF, 2000) and the World Health Organization (WHO, 1991) [21]. For pediatric patients, the prescribed CARV doses for infants and children under 12 years old range from 0.05 to 0.10 mg/kg of the child’s weight every 12 h at the beginning of treatment [22]. Considering that CARV concentration at our formulation is 1 mg/mL, the concentration of sucralose does not exceed its ADI.

With the aim of further masking the taste of the liquid formulations, some flavorings (white chocolate, strawberry, apple pear, lemon, orange juice, and cola) were added to the CARV formulations already containing sucralose.

### 2.2. In Vivo Palatability Tests

#### 2.2.1. Testing Protocol

The taste masking of bitter drugs represents a fundamental challenge during the preparation of oral liquid formulations, as most drugs are unpalatable, influencing patient acceptance. We performed a palatability test on human volunteers by adopting a responsive sensory approach to facilitate the development of a palatable pediatric CARV formulation. This study was performed in two sessions of 1 h duration, and eighteen formulations in total were assessed. Multiple sensory evaluation panels were conducted to measure key palatability attributes through two questionnaires (Supplementary File S1 and S2).

Fifteen plus thirteen healthy human volunteers participated in this single-blind, prospective, single-center study using the “swirl and spit” method. This study was executed according to the Helsinki Declaration for bio-medical research, including human and Good Clinical Practice (GCP) rules [23]. The protocol of this study was reviewed and approved by the Institutional Review Board of the Ethics Committee for Research at Bellvitge University Hospital (Barcelona, Spain) (ID Number PR103/22) (Supplementary File S3). All the enrolled volunteers signed a written informed consent after receiving the required information about the study.

Participants recruited were healthy adult volunteers between 21 and 62 years of age. Fifteen participants (9 females and 6 males) were enrolled for the first questionnaire as well as thirteen participants (7 males and 6 females) for the second questionnaire. This sample size was considered sufficient to perform a statistical analysis to detect the differences among the dispersions from the inter-subject variabilities.

Subjects were excluded from the study if they met any of the following criteria:

− Subject under the age of 18. The use of adult human panels is justified on the grounds of ethical concerns with the direct use of children as taste panels.
− Pregnant or lactating women.
− Allergies, hypersensitivities, or intolerance to carvedilol, malic acid, sucralose, and any flavoring agent of study formulation.
− Subjects with an active infectious process at the time of inclusion, whether chronic or acute
− Subjects with any condition that may affect the sense of taste or smell (such as a respiratory infection, febrile illness, mucositis) or who are taking medication that could influence it.

Each volunteer was asked to fill out the first sensory questionnaire (Supplementary File S1), in which they evaluated ten formulations (A1 to A6 and B1 to B4) and eight formulations in the second questionnaire (C1 to C8) (Supplementary File S2). Samples were anonymized (single-blind) and presented in a randomized order. Participants were instructed to swirl 10 mL of the test samples around their mouths for 5 s, ensuring thorough contact between the test sample and the oral surfaces inside the mouth before providing ratings. Immediately after expectoration, participants assessed the odor and taste of each formulation using a questionnaire equipped with categorical and quantitative scales. To neutralize any aftertaste, participants rinsed their mouths with water and, if necessary, consumed plain crackers both before and after each sample.

Participants were individually positioned with adequate distance to prevent interaction, ensuring a quiet and pleasant environment. Prior to the investigation, participants were allowed a neutral meal (non-spiced, lightly salted) at least 30 min beforehand. Volunteers who smoked were required to abstain from smoking for at least one hour preceding the tests.

Typical adverse events with an emphasis on known gastrointestinal and central nervous system symptoms were followed over 72 h through a confirmation email.

#### 2.2.2. CARV Formulations Included in the Study

CARV formulations we studied herein were based on the optimized CARV formulation described in our last research [10]. The characteristics of the CARV formulation we developed are presented in Table 2. To prepare the CARV solution, we weighed, transferred, and dissolved 0.8 g/100 mL of malic acid into a beaker containing purified water. A sufficient amount of CARV pharmaceutical grade was added to each medium at 1 mg/mL and mixed via magnetic stirring until complete dissolution. We prepared a reference formulation for the palatability study, coded as A1, using this method. To prepare the remaining formulations, the established amount of sucralose was added and mixed until complete dissolution. Subsequently, flavoring agents were added in the same manner to the corresponding formulations. 

##### Tests 1 and 2

Firstly, Tests 1 and 2 were performed together through the first questionnaire of 1 h duration, and ten formulations were assessed (A1 to A6 and B1 to B4). Formulations included in these tests and their detailed composition are shown in Table 3 and Table 4. The aim of Test 1 was to select a preferred flavoring by the participants, whereas that of Test 2 was to determine the minimum dose of sucralose acceptable for the participants.

A1 did not contain any sweetener or flavoring agent; A2 only contained sucralose at a low concentration (0.05%); A3 to A6 contained sucralose (0.05%) added to a different flavoring at 0.20%. B1 to B4 contain different quantities of sucralose, from 0.02% to 2.0%. All formulations included in the palatability study were prepared and presented at room temperature the day before the tests.

##### Test 3

C1 to C8 were prepared to perform a second questionnaire within the palatability study based on Tests 1 and 2 results. C1 and C8 contained 0.2% of sucralose; C2 to C8 also contained one or two flavoring agents at a maximum of 0.2% in total. Their detailed composition is presented in Table 5.

#### 2.2.3. Outcome Measures and Analysis of Results

All the enrolled volunteers were asked to record different palatability attributes of every formulation using a questionnaire with categorical and quantitative scales after spitting each test sample. Questionnaires for Test 1 to Test 3 consisted of different sections depending on the assessed palatability attribute. After providing scores for individual senses, participants were asked to rank each product. An overview of Test 1 to Test 3 outcome measures, terms of acceptability, and objectives are shown in Table 6.

##### Taste, Aftertaste, and Odor Pleasantness

Each sample’s odor and taste were assessed as the degree of pleasantness, recorded as the rated taste intensity or score, using the questionnaire on a 4-point Likert scale from “very unpleasant (1)” to “very pleasant” (4). The scale of taste and odor intensities was as follows: 1 = very unpleasant, 2 = unpleasant, 3 = pleasant, 4 = very pleasant. Additionally, aftertaste was reported 1 min after spitting out the sample. Firstly, participants were asked if they perceived an aftertaste. Consequently, participants who confirmed an aftertaste assessed the aftertaste’s degree of pleasantness using the questionnaire on a 4-point Likert scale from “very unpleasant (1)” to “very pleasant” (4).

Results of the samples’ odor and taste degree of pleasantness for participants were turned from a 4-Linkert scale into a numerical 0–100 scale. Samples with odor, taste, and aftertaste rated 0–50 were regarded as not acceptable palatability, and those rated 50–100 were of acceptable palatability.

##### Palatability Scores and Taste-Masking Abilities

The test ended with a palatability rating for each sample using a numerical scale from 0 to 10. Ratings of 0 to 5.0 were regarded as “not acceptable palatability”, and ratings of 5.0 to 10 as “acceptable palatability”. In addition to these responses, participants were also given the option to provide additional qualitative written comments relating to their organoleptic perception of each sample. 

Taste-masking was assessed as the ability of the optimized formulation to have a significantly reduced bitterness, as well as an increase in taste/aftertaste/odor pleasantness and palatability score compared with the reference formulation (A1). 

Microsoft Excel was used to calculate these mean scores, standard deviations, medians, quartiles mean, and quartile coefficient of dispersion (QCD).

##### Sweetness Level

B1 to B4 formulations contained different quantities of sucralose. The aim of this test was to find the optimal quantity of sucralose for the participants as part of CARV formula optimization. B1 to B4 sweetness levels were assessed using a numerical scale from 0 to 10 included in the sensory questionnaire. The optimal value of sweetness was considered around 5.0. A sweetness value < 5.0 was evaluated as mild, as well as a sweetness value > 5.0 was evaluated as moderate. The level of sweetness converted to a numerical scale of 0 to 10 is presented in Table 7.

##### Taste Attributes

Furthermore, participants rated taste attributes and perceptible taste characteristics for all the samples with intensity values to establish a sensory taste profile. Taste attributes assessed were bitterness, sourness, sweetness, and if the general taste was strong and/or delicate.

##### CART (Classification and Regression Tree) and PCA (Principal Components Analysis)

This exploratory study was descriptive in nature; thus, we performed descriptive statistics of patient characteristics, including sex and age. A 4-node CART analysis was performed in order to obtain the Relative Variable Importance for the final palatability score (another variable). The studied variables were taste pleasantness, taste identification, smell pleasantness, smell identification, age, and sex/gender.

Furthermore, PCA was conducted to reduce the number of variables in the data set, as well as preserve as much information as possible. PCA changed the coordinates by means of the exchange of the original variables by linear combinations of them. 

CART and PCA results were provided by Minitab 21.0 version (Minitab, LLC, State College, PA, USA).

### 2.3. Stability Study

The physical, chemical, and microbiological stability of the highest-rated formulation by the participants through the three palatability tests was studied for 6 months under various conditions. Three batches of this formulation were prepared, packaged in 30 mL amber bottles, and stored at temperatures of 25 °C, 30 °C, and 40 °C. As CARV degradation is susceptible to exposure to ambient light in a solution state, the CARV formulations were stored in bottles capped with amber covers to prevent light exposure. Samples from each batch were collected at different intervals (1, 3, and 6 months) and subsequently analyzed in duplicate for pH levels, appearance, and CARV assay following the International Conference on Harmonization (ICH) guidelines [24].

The assessment of appearance aimed to confirm uniformity and the absence of any precipitation. Both appearance and pH levels were assessed using samples directly extracted from the bottle. pH measurements were conducted using a pH meter (HANNA Instruments, Guipúzcoa, Spain) to detect any potential alterations in the solutions. 

Chemical stability was considered acceptable if the drug concentration remained above 95.0% of the initial concentration. CARV percent assay with respect to initial time was evaluated using the validated HPLC method detailed in this section. Prior to conducting the CARV assay, each sample was filtrated using a syringe filter featuring a pore size of 0.45 µm (Agilent, Barcelona, Spain). Subsequently, these samples were diluted with the mobile phase at a 1:10 dilution to quantify the CARV content through HPLC.

Specifications for each parameter are as follows:

- Appearance: clear solution, translucent, without any undissolved particles.
- pH: initial pH ± 0.2.
- CARV assay (%): 95–105.

Chromatographic separation of CARV was carried out using a Luna C18 column sized 150 mm × 4.6 mm, with a particle size of 5 µm, made of stainless steel (Phenomenex, Barcelona, Spain). The mobile phase constituted HPLC-grade acetonitrile and buffer solution (pH 4.5, potassium dihydrogen phosphate) in an isocratic program (35:65 ratio, respectively). A flow rate of 1 mL/min was maintained. Detection was performed using a DAD detector set at 240 nm, and 5 µL was injected for analysis. The HPLC analysis was conducted at a temperature of 40 °C, with each determination requiring approximately 30 min. This method aligns with the one outlined in the European Pharmacopoeia 11th edition [25].

Microbiological tests were performed according to the methods described in European Pharmacopoeia 11.2 [26,27,28]. Representative samples of the formulation were analyzed initially and at a 6-month time point and stored at 25 °C, 30 °C, and 40 °C.

Moreover, a 15-day in-use stability for the best-rated formulation in the palatability study was performed. We prepared another batch of this highest-rated formulation, packaged it in a 100 mL amber bottle, and stored it at 25 °C for a 15-day in-use stability study. This bottle was open daily throughout the storage period since 1 mL of the formulation was removed with a syringe appropriate for pediatrics (dosing device). Additionally, a suitable quantity of the formulation was tested for pH, appearance, and CARV assay at 0, 5, 10, and 15 days. Microbiological tests were also performed at initial and final time points. Physical, chemical, and microbiological tests were performed according to ICH Q1A(R2) [24]. The EMA has incorporated ICH guidelines into its recommendations for conducting in-use stability studies. The primary concern of the EMA was the stability of pharmaceutical products in a multi-dose container after its initial opening, considering that the repeated opening and closing of the container during its use could impact its physicochemical or microbiological properties. Ideally, the study setup should simulate the actual usage of the product in clinical practice to the greatest extent possible [29]. 

## 3. Results

### 3.1. Palatability Study

The taste assessment results of studied CARV formulations for all the participants were collected from the questionnaires, and they are explained below. No drop-out occurred, and all subjects completed the full questionnaire. No adverse reaction was reported throughout the study.

#### 3.1.1. Test 1: Taste and Odor Pleasantness; Palatability Scores

**Figure 1:**
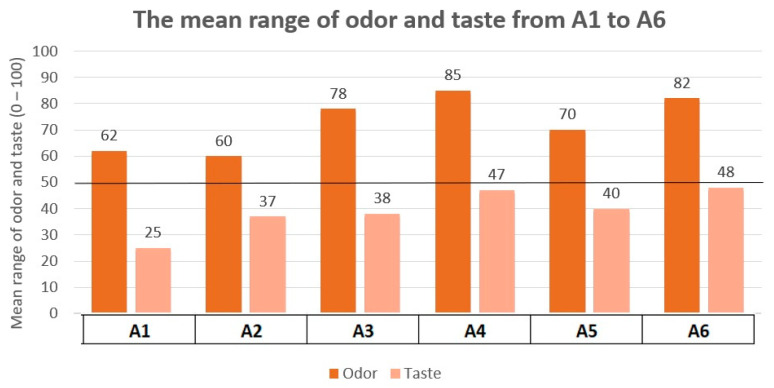
Taste and odor pleasantness A1–A6 results. Test 1.

Results of the mean taste and odor pleasantness for formulations A1 to A6, as evaluated through Test 1, are shown in Figure 1. Results of A1 to A6 palatability score for participants (*n* = 15) are outlined in Table 8. Regarding odor pleasantness, all evaluated formulations surpassed the minimum target of 50. Odor received better ratings, both in formulations with low taste ratings and high ones. Therefore, it was not significant, but it should at least reach a score of 50. To interpret the taste results, “pleasantness” and “palatability score” should be considered. The palatability score was evaluated on a 0 to 10 scale, with 5.0 being the acceptable score. The reference formulation (A1) obtained a taste pleasantness score of 25. None of the formulations A1 to A6 achieve a “pleasantness > 50” rating for taste, nor a “palatability score” > 5.0, making none of them acceptable. Formulations closest to acceptability are A4 and A6, with scores of 47 and 4.91 and 48 and 4.63, respectively, although this was not acceptable. Therefore, it was necessary to conduct another test (Test 3) with new flavorings, a different concentration of sucralose, or a combination of these, considering the results obtained in the interpretation of Test 1 and Test 2.

#### 3.1.2. Test 2: Sweetness Level

Test 2, which comprised formulations B1 to B4, was conducted to determine the optimal sucralose concentration according to the participants. Results of B1 to B4’s sweetness level for the participants are presented in Table 9. None of the formulations B1 to B4 reached the required optimal sweetness level of 5.0. Nevertheless, formulation B4 came closest to the optimal level with a sweetness value of 3.73. These results were evaluated to carry out Test 3, which includes formulations C1 to C8 with a concentration of sucralose of 0.20%, the same as B4.

#### 3.1.3. Test 3: Taste and Odor Pleasantness; Palatability Scores

**Figure 2:**
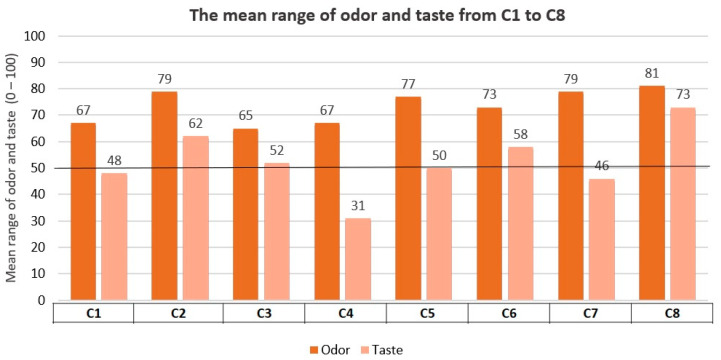
Taste and odor pleasantness results: C1–C8. Test 3.

Results of the mean taste and odor pleasantness for formulations C1 to C8 are shown in Figure 2, whereas results of the C1 to C8 palatability score for participants (*n* = 13) are expressed in Table 10. These results were evaluated through Test 3. Several formulations in Test 3 exceeded a score of 50 on the “taste pleasantness” scale (C2, C3, C5, C6, and C8) (Figure 2). Also, several formulations in this test scored above 5 on the “palatability score” (C1, C2, C3, C5, C6, and C8) (Table 10). Regarding odor pleasantness, C1 to C8 formulations surpassed the minimum target of 50.

#### 3.1.4. Tests 1 and 3: Aftertaste Pleasantness

**Figure 3:**
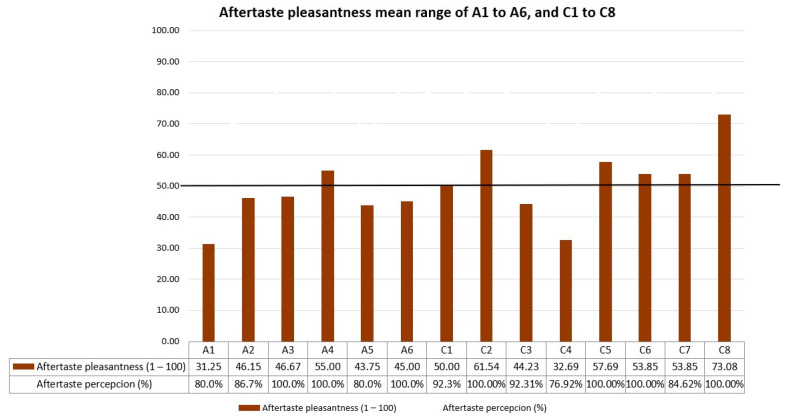
Aftertaste pleasantness results of A1 to A6 and C1 to C8 formulations.

Aftertaste pleasantness mean range of all formulations evaluated through Test 1 and Test 2 are outlined in Figure 3. The acceptability level for aftertaste was also set at 50. Only formulation A4 from Test 1 exceeded the acceptability threshold of 50 for the “aftertaste”. Conversely, several formulations from the Test 3 surpassed this threshold (C1, C2, C5, C6, C7, and C8). Formulation C8 led this ranking with a score of 73.08, with 100% of the participants perceiving this aftertaste.

#### 3.1.5. Tests 1 and 3: Combined Palatability Scores

**Figure 4:**
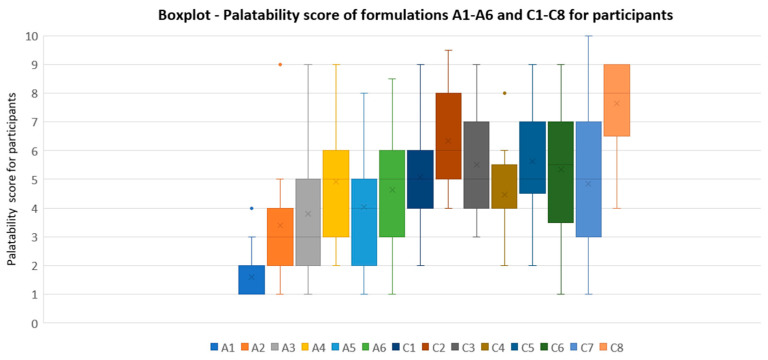
Boxplot representing palatability score results of A1 to A6 and C1 to C8 formulations.

Palatability scores for each of the fourteen tested formulations by the participants are displayed in Table 8 and Table 10. The boxplot depicted in Figure 4 illustrates palatability scores for A1 to A6 and C1 to C8. Mean scores, standard deviations, medians, quartiles mean, and quartile coefficient of dispersion (QCD) are also presented in Table 11.

A quartile coefficient of dispersion (QCD) value close to 1 indicates more dispersion of the results, whereas a QCD close to 0 indicates less variability between the measures. C8 and C4 exhibited the minimum QCD among all samples studied, 0.16, which indicates a low variability between their results.

None of the formulations in series A achieved a palatability score rated >5.0, prompting the continuation of the research by preparing the formulations in series C. Nevertheless, A4 and A6 were the best-rated in this series, with scores of 4.91 and 4.63, respectively. Within the C series, several formulations exceeded the acceptability threshold of 5.0 (C1, C2, C3, C5, C6, C8), with C8 standing out with a palatability score of 7.65.

#### 3.1.6. Tests 1 and 3: Taste Attributes

**Figure 5:**
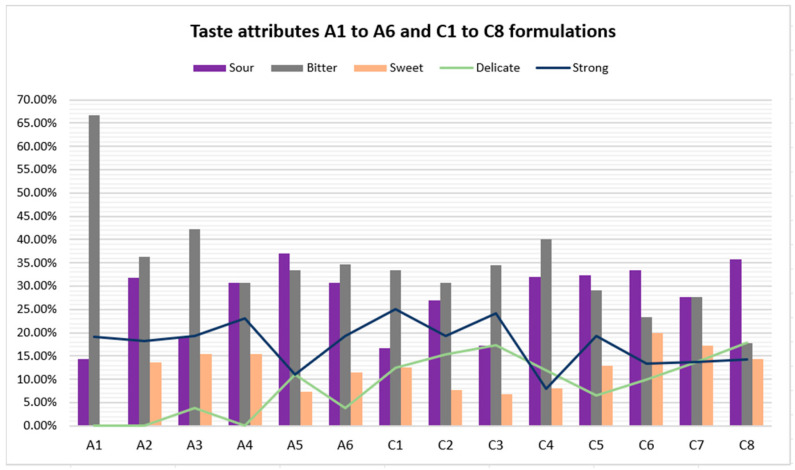
Taste attributes results of A1 to A6 and C1 to C8 formulations.

Taste attribute results of formulations A1 to A6 and C1 to C6 are shown in Figure 5, Table 12 and Table 13. Reference formulation (A1) received 66.67% bitterness and 0.0% sweetness ratings from the participants. The bitterness level was reduced with A2 (sucralose 0.05%) to 36.36%, and sweetness increased to 13.64% compared to A1.

Considering the results of formulations included in Test 1 (A1 to A6), the formulation rated with the lowest bitterness percentage was achieved by A4 with 30.77% bitterness. Conversely, formulations evaluated in Test 3 (C1 to C8) obtained better bitterness results since several formulations outperformed the bitterness level of A4: C5 (29.03%), C6 (23.33%), C7 (27.59%), and C8 (17.86%). Formulation C8 topped the rankings, as the bitterness level decreased from 66.67% (A1) to 17.86% (C8), while the sweetness level increased from 0.0% (A1) to 14.29% (C8). Furthermore, this is the only one of the fourteen formulations that received a higher “delicate” score (17.86%) compared to “strong” (14.29%) in describing the overall taste. Regarding sourness, the addition of sucralose and/or flavoring agents elevates its level, as it increases from 14.29% (A1) to 35.78% (C8).

#### 3.1.7. Test 3: PCA and CART Results

PCA and CART analysis were only carried out for formulations included in the last Test 3, C1 to C8. A 4-node CART analysis was performed to obtain the Relative Variable Importance of all variables for the final palatability score variable. Variables studied during the taste assessments were age, sex or gender, smell identification (Smell-ID), smell pleasantness (Smell-like), taste identification (Taste-ID), taste pleasantness (Taste-like), and final palatability score.

**Figure 6:**
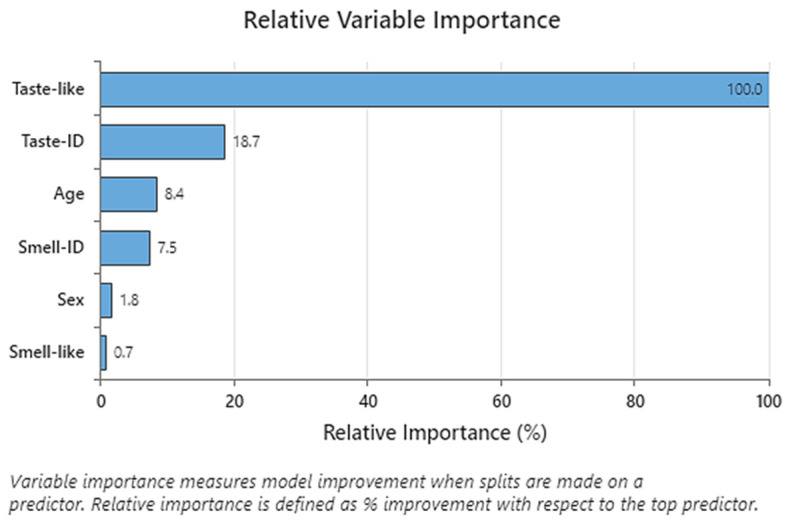
CART results: Relative Variable Importance (%). Variables: Age, Smell-ID (smell identification), final palatability score, Taste-like (taste pleasantness), Taste-ID (taste identification), and Smell-like (smell pleasantness).

The results of the CART analysis following the variables’ importance order are shown in Figure 6. Taste pleasantness was highly related to the final palatability score (100% relative importance), keeping taste identification in the second position with 18.7% relative importance. Smell identification was not considered due to its low relative importance.

**Figure 7:**
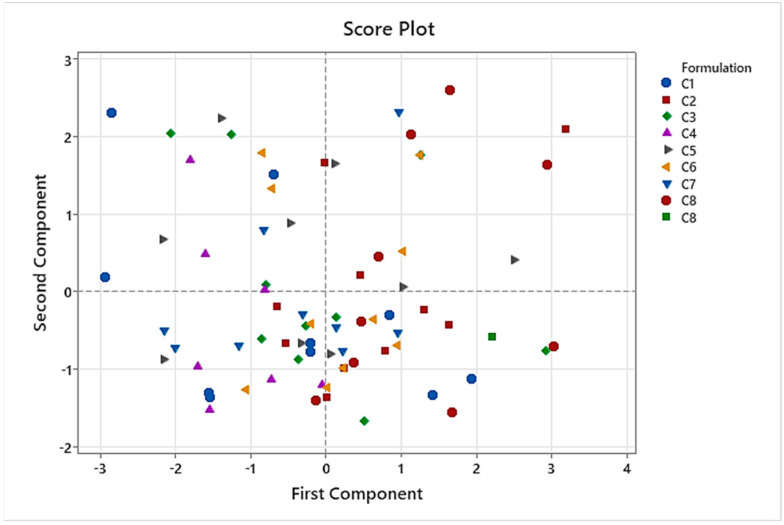
Results of PCA. Score Plot representing C1 to C8 formulations.

Furthermore, the new coordinate system provided by the PCA had the property of orthogonality, meaning independence between each principal component. The correspondence between the original variables and principal components is shown in Table 14. PC1 (First Component) and PC2 (Second Component) were chosen to be represented in the Score Plots (Figure 7).

**Figure 8:**
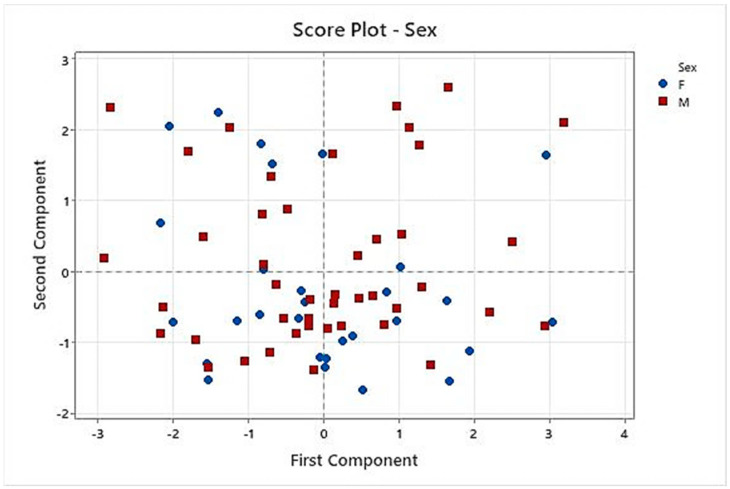
Results of PCA. Score Plot representing gender results.

**Figure 9:**
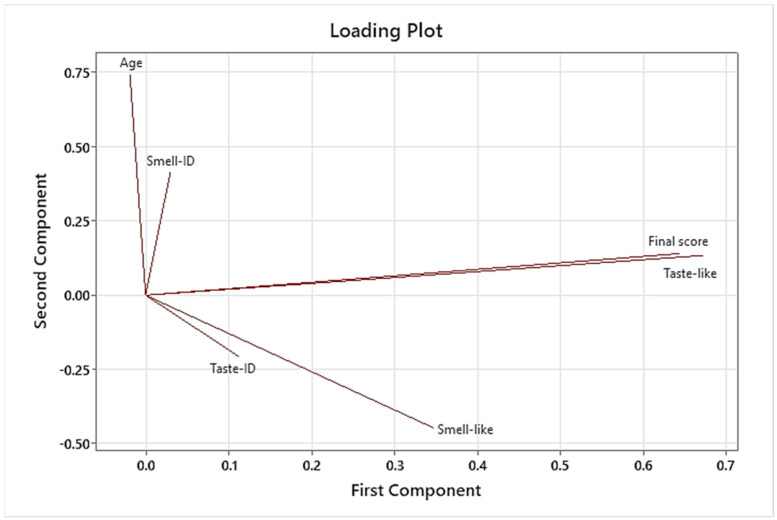
Results of PCA: Loading Plot of all studied variables. Variables: Age, Smell-ID (smell identification), final palatability score, Taste-like (taste pleasantness), Taste-ID (taste identification), and Smell-like (smell pleasantness).

The Score Plots (Figure 7 and Figure 8) show where the individual points are located in terms of the new coordinate system. Proximity points in Score Plot (Figure 7) mean similar properties, while larger separation should be understood as little or no similarities. When depicting formulations with different shapes/colors, neither clusters nor any specific formulation have characteristics that stand out. However, formulation C8 is presented in the first and quarter quadrants only, while formulation C4 shows the opposite behavior, standing in the second and third quadrants. The meaning of any dot position is related to the direction of the dimension growth, just like the direction of each variable in the Loading Plot (Figure 9). Those individuals lying to the right part of the Score Plots have higher taste pleasantness and final palatability scores. Those in the fourth quadrant are more influenced by smell pleasantness. Both opposite directions to the mentioned ones show where negative appreciations were observed (left part of the Score Plots). As already mentioned in the PCA study results, taste pleasantness was the most related to the final palatability score variable. 

Considering gender as metadata for this study, the Score Plot (Figure 8) is colored red and blue for males and females, respectively. No association or cluster was observed, so results were not affected by gender in this study.

### 3.2. Stability Study

Considering the results from Tests 1, 2, and 3, comprising the entire palatability test, the formulation most highly rated by the participants, C8, was chosen as the optimized CARV palatable formula. Therefore, C8 was included in a 6-month stability study at three storage conditions. We prepared three batches of a new C8 formulation coded as C8_P2 and included it in the stability study. Every batch was analyzed in duplicate at each stability time. The poolability of results from the three batches, batch × time interaction, was previously verified with a significance level *p*-value > 0.25. Therefore, results data from the three batches can be combined. Table 15 presents CARV assay results for C8_P2. All results were according to the 95.0–105.0% specification.

The initial C8_P2 pH value was 2.47, and initial data on the C8_P2 appearance indicated that it was a clear, translucent solution without any undissolved particles. C8_P2 exhibited identical physicochemical characteristics to those of the reference CARV solution on which it is based [10]. pH results for C8_P2 were highly stable over time with a range of variation ± 0.03. They are not represented for these reasons. C8_P2 appearance conformed and kept to the specifications at each time studied.

Microbiological results after 6-month storage were in accordance with the requirement for non-sterile oral substances for pharmaceutical use in European Pharmacopoeia 11.2 [26]. Total aerobic microbial count (TAMC) and total combined yeasts/mold (TYMC) count were inferior to the limit of 103 CFU/mL and 102 CFU/mL, respectively. The absence of Escherichia coli in 1 g of formulation was also confirmed.

The in-use stability results of one batch of C8-P2 for 15 days at 25 °C are shown in Table 16 and Table 17. Appearance and pH did not undergo any variation. CARV assay results were according to the 95.0–105.0% specification. Microbiological results for the initial and final point time were also according to the European Pharmacopoeia specification [26]. C8-P2 formulation satisfied the requirements for European Pharmacopoeia after 15 days of storage at 25 °C considering the simulation of once-daily administration.

## 4. Discussion

The next step to optimize the CARV liquid formulation we have developed for pediatrics [10] was to make it more palatable in order to increase its acceptability in this population. Most APIs have an unpleasant taste when dissolved in water, so finding a method to mask each API’s taste is a challenge. CARV in solution also exhibits an unpleasant bitter taste; thus, the development of a palatable CARV liquid formulation was considered essential for children, especially those under 7 years old, since they are not able to swallow solid pharmaceutical forms [30]. 

Acceptability of pediatric pharmaceutical formulations holds immense importance for children, given that treatment compliance heavily relies on an acceptable taste. Hence, ensuring adequate palatability of oral liquid formulations becomes an important factor in their acceptability, where flavors may be essential to achieve this objective [1,3]. Moreover, in enhancing the palatability of pediatric formulations, it is crucial to deliberate on excipients that are safe for this extremely young population, considering that this treatment could be administered very early in life, possibly even at birth [31].

Different approaches might be utilized to obscure the unpleasant taste of APIs in pediatric oral dosage forms. In our case, sweeteners and flavors were added to CARV liquid formulations to mask the bitter taste of CARV. Syrups are commonly employed as carriers for compounded oral formulations. However, their high sucrose content makes them unsuitable for pediatric patients with diabetes and hereditary fructose intolerance. Moreover, sucrose alters dental plaque pH, leading to tooth enamel dissolution and contributing to dental cavities [2]. Cherry and strawberry flavors, in combination with a high-intensity sweetener, may suit the United States and European pediatric market. Other typical flavors used in pediatric formulations include orange, vanilla, and grapefruit. For the selection of the most suitable flavor for a pediatric medication, the taste to cover (acid, alkaline, bitter, salty, or sweet) must be considered. To cover a bitter taste, most flavors used are cherry, chocolate, grapefruit, licorice, strawberry, peach, raspberry, and tutti-frutti [1].

Palatability was selected as the criterion of choice for a new palatable age-adapted formulation, so we considered taste evaluation as a key step during the development of pediatric oral formulations. In our research, different flavorings, or a combination of two, in addition to a sweetening agent (sucralose), were chosen to create CARV palatable solutions. These formulations were then put through a taste test involving human adult volunteers to assess how effectively these formulations could cover up the unpleasant taste of CARV when compared to the reference CARV solution [10]. This study aimed to identify the CARV solution that received the highest palatability score and the lowest bitterness level among all healthy human volunteers who participated in the study. One limitation of this study is that the human taste panel was conducted in healthy adults rather than children (who represent the target population) for a subsequent extrapolation of the results. As it is mentioned, for ethical reasons, conducting a clinical trial in children is quite complex [19]. 

Taste evaluation was made up of three tests. Test 1 and Test 2 were performed in order to find the best acceptable flavoring for the participants to mask the unpleasant taste of CARV, as well as the best acceptable concentration of sucralose. Flavoring agents used in Test 1 were the ones highly recommended by the EMA [1], which explains why they were the starting point. However, the results of Test 1 revealed that these flavorings were not suitable for masking the unpleasant taste of CARV. Conversely, results from Test 2 showed the optimal concentration of sucralose. Due to these outcomes, Test 3 was conducted in a second session, which included formulations with the optimal concentration of sucralose and different flavoring agents or a combination of these.

Formulations tested in Test 3 (C1 to C8) obtained significantly better results for participants compared to formulations tested in Test 1 (A1 to A6). Formulations closest to acceptability in Test 1 are A4 (strawberry 0.2% and sucralose 0.05%) and A6 (apple pear 0.2% and sucralose 0.05%), with scores of taste pleasantness and palatability for participants of 47/100 and 4.91/10, and 48/100 and 4.63/10, respectively. Nevertheless, these scores did not exceed our established acceptability limit.

Concerning sweetness-rated results for B1 to B4 formulations, B4 was the closest to the required optimal level (5/10). Therefore, the amount of sucralose in formulation B4 (0.20%) was chosen for formulations comprised in Test 3 (C1 to C8). It was not possible to increase the sucralose level in the formulation as it would exceed the allowable ADI [21].

Referring to the palatability scores results of formulations in series C, one formula stood out for its high values in all the tests performed: C8 (lemon 0.15%-cola 0.05% flavorings, and 0.20% sucralose). Scores of C8 in the test were 73/100 for taste pleasantness, 81/100 for odor pleasantness, 73/100 for aftertaste pleasantness, and 7.65/10 for final palatability. All these scores exceeded our acceptability limits, making C8 the best palatability-rated formula among the three palatability tests conducted. Final palatability scores for participants increased from 1.6 (A1, reference CARV solution) to 7.65 (C8), exhibiting the taste-masking ability for C8.

The boxplot presented in Figure 4 illustrates palatability scores for A1 to A6 and C1 to C8. This boxplot clearly demonstrates that all the formulations in series C with the incorporated improvements are better rated than the initial ones in series A. Additionally, C8 was the formulation with lower variability results between the participants, exhibiting the minimum QCD among all samples studied of 0.16.

These improvements were the increase in sucralose from 0.05% (series A formulations) to 0.20% (series C formulations), in addition to new flavorings, such as lemon and cola flavors, or a combination of two agents. The high palatability score of C8 for the participants could be explained by the synergistic effect of combining sucralose with these sweetening agents, in addition to the specific concentration of these.

Furthermore, formulations in series C obtained better bitterness results. Once again, formulation C8 topped the rankings, as the bitterness level decreased from 66.67% (A1 reference CARV solution) to 17.86% (C8). Concerning the sourness level, C8 achieved a percentage of 35.78%. Despite having a high sourness percentage, it did not cause unpleasantness among the participants, given that C8 is the highest-rated formulation among the fourteen. Hence, the bitterness level of CARV formulations studied significantly decreases with an increase in the amount of sucralose in the formulation and even more with an appropriate combination of sucralose and one or two flavoring agents.

CART analysis performed on C1 to C8 formulations showed that taste pleasantness was highly related to the final palatability score, so we especially considered these variables for the taste assessment. Results of PCA demonstrated that C8 had the best results for these two variables because C8 spots were presented in the quadrants influenced by taste pleasantness and final palatability score. Another conclusion of PCA was that the results of the taste assessment we performed were not affected by gender in this study.

These results suggested that sucralose, as a sweetener, in addition to lemon and cola as flavoring agents, would effectively minimize the bitterness of CARV in its solution state. The achieved masking of CARV’s unpleasant taste properties presents a tangible advantage for its oral administration, especially in pediatric use, thereby enhancing children’s compliance with the pharmaceutical formulation.

Furthermore, through a stability study according to ICH guidelines [24], C8 has proven to be physically, chemically, and microbiologically stable after 6 months of storage at 25, 30, and 40 °C. Moreover, in-use stability results of C8 were confirmed for 15 days at 25 °C. We utilized Minitab 21.0 to analyze the CARV assay results (%) from three stability batches of C8_P2 over a 6-month period to estimate the formulation’s shelf life. The correlation observed between CARV assay (%) and time formed a straight line with a zero slope (~100% adjustment), suggesting no substantial variation in CARV assay (%) throughout the study duration. Consequently, these findings were employed to extrapolate a stability duration of 12 months, aligning with guidelines from ICH Q1(a). Simultaneously, C8_P2 continues undergoing a long-term stability study for 24 months at 25 and 30 °C to verify these results. All data accomplished the upper and lower specifications.

The C8_P2 formulation herein developed, a 1 mg/mL sweetened and flavored oral CARV aqueous formulation, had good stability according to the CARV assay (%), pH, and appearance results and satisfied the requirements of European Pharmacopoeia. It can be concluded that we developed an optimized CARV 1 mg/mL palatable solution, especially interesting for patients with swallowing difficulty in elderly populations, in addition to challenges in acceptability as pediatric patients [1].

## 5. Conclusions

In conclusion, CARV, an optimized palatable solution, was successfully obtained through a “swirl and spit” taste assessment in human volunteers. This formulation was the highest rated CARV solution by the participants, C8, which contains a combination of lemon (0.15% *w*/*w*) and cola (0.05% *w*/*w*) flavors, in addition to sucralose (0.20% *w*/*w*), as a sweetener. The palatability results of C8 showed that this masking method reduced the bitterness of CARV from 66.67% (reference CARV solution) to 17.86% (optimized CARV solution). CART and PCA analysis were performed to evaluate the most relevant variables to consider for the taste assessment. These variables were taste pleasantness and final palatability score, whose results for the optimized formulation were 73/100 and 7.65/10, respectively. Moreover, the stability studies indicated that the optimized CARV formulation was stable for 6 months at 25, 30, and 40 °C and for 15 days at 25 °C in the case of a multi-dose container after its first opening. Aiming to address the palatability challenges encountered by pediatric patients, we developed a palatable CARV formulation appropriate for them. Our formulation distinguishes itself by a unique combination of excipients, namely sucralose as a sweetener and cola-lemon as a flavoring agent, which favorably enhances CARV’s bitter taste. According to the literature, it is the first attempt to prepare CARV-palatable solutions appropriate for pediatrics.

## Figures and Tables

**Table 1:** Flavoring agents included in the palatability study.

Flavoring Agent	Supplier Code	Supplier	Batch
White chocolate	LAB19462	Kerry	8900161472
Strawberry #1	F-10815	Kerry	8900161471
Strawberry #2	S-139770	Givaudan	D9A019922
Apple Pear	S-144937	Givaudan	D90179749
Lemon #1	S-080551	Givaudan	D9G0000063
Lemon #2	L-249791	Givaudan	D9A0202620
Orange juice	75935-71	Givaudan	D9A0208621
Cola	CS-492-239-7	Givaudan	D9A0198089

**Table 2:** Characteristics of the optimized CARV formulation developed.

CARV concentration	1.0 mg/mL
Malic acid concentration	0.8 g/100 mL
Solvent	Purified water
Aspect	clear solution, translucent, without any particles undissolved
pH value	2.5 to 3.0

**Table 3:** Composition of A1–A6 formulations (Test 1).

Formula	CARV (mg/mL)	Malic Acid (% *w*/*w*)	Sucralose (% *w*/*w*)	Flavoring	
				Flavoring Agent	% *w*/*w*
A1 (reference)	1.0	0.8	-	No flavoring	
A2	1.0	0.8	0.05		
A3	1.0	0.8	0.05	White chocolate	0.2
A4	1.0	0.8	0.05	Strawberry #1	0.2
A5	1.0	0.8	0.05	Strawberry #2	0.2
A6	1.0	0.8	0.05	Apple pear	0.2

**Table 4:** Composition of B1–B4 formulations (Test 2).

Formulation	CARV (mg/mL)	Malic Acid (% *w*/*w*)	Sucralose (% *w*/*w*)	Flavoring
B1	1.0	0.8	0.02	No flavoring agent
B2	1.0	0.8	0.05	
B3	1.0	0.8	0.1	
B4	1.0	0.8	0.2	

**Table 5:** Compositions of C1–C8 formulations (Test 3).

Formulation	CARV (mg/mL)	Malic Acid (% *w*/*w*)	Sucralose (% *w*/*w*)	Flavoring		
				Flavoring Agent		% *w*/*w*
C1	1.0	0.8	0.20	No flavoring		
C2	1.0	0.8	0.20	Lemon #1		0.1
C3	1.0	0.8	0.20	Orange juice		0.1
C4	1.0	0.8	0.20	Lemon #2		0.2
C5	1.0	0.8	0.20	Apple Pear		0.2
C6	1.0	0.8	0.20	Flavoring 1	Lemon #2	0.15
				Flavoring 2	Orange juice	0.05
C7	1.0	0.8	0.20	Flavoring 1	Lemon #2	0.15
				Flavoring 2	Apple pear	0.05
C8	1.0	0.8	0.20	Flavoring 1	Lemon #2	0.15
				Flavoring 2	Cola	0.05

**Table 6:** Overview of test 1 to test 3 outcome measures, terms of acceptability, and objectives.

	Outcome Measures	Terms of Acceptability	Objectives
**Questionnaire 1 (day 1)**			
**Test 1** A1 to A6 formulations	(1) Taste, aftertaste, and odor pleasantness	Odor, taste, aftertaste rated > 50.0	Select a preferred flavoring by the participants
	(2) Taste attributes	Significant reduction of bitterness compared to reference	
	(3) Taste masking and palatability scores	Palatability score > 5.0	
**Test 2** B1 to B4 formulations	(1) Sweetness level	Around 5.0 (optimal value)	Determine the minimum dose of sucralose acceptable
**Questionnaire 2 (day 2)**			
**Test 3** C1 to C8 formulations	(1) Taste, aftertaste, and odor pleasantness	Odor, taste, aftertaste rated > 50.0	Find the best combination of flavoring/sucralose rated by the participants
	(2) Taste attributes	Significant reduction of bitterness compared to reference	
	(3) Taste masking and palatability scores	Palatability score > 5.0	

**Table 7:** Sweetness level converted to a numerical scale of 0 to 10.

0	1.0	2.0	3.0	4.0	5.0	6.0	7.0	8.0	9.0	10
unidentifiable	(+) mild (−)				optimal	(−) moderate (+)				overwhelming

**Table 8:** Results of A1 to A6 palatability score (*n* = 15). Test 1.

Formulation	A1	A2	A3	A4	A5	A6
Palatability score (mean ± SD)	1.6 (±0.99)	3.4 (±1.99)	3.8 (±2.18)	4.91 (±2.1)	4.03 (±2.17)	4.63 (±2.16)

**Table 9:** Results of B1 to B4’s sweetness level for the participants (*n* = 15). Test 2.

Formula	B1	B2	B3	B4
Sweetness level (mean for participants ± SD) (*n* = 15)	1.4 (± 2.38)	2.2 (± 1.86)	3.07 (± 1.39)	3.73 (± 2.15)
	Very mild	Mild	Mild	Mild close to optimal
Conclusion	Not acceptable	Not acceptable	Not acceptable	Acceptable. 5.0 optimal value was not reached, but it is not recommended to add >0.2% of sucralose in pediatrics [21].

**Table 10:** Palatability score results C1 to C8 (*n* = 13). Test 3.

Formulation	C1	C2	C3	C4	C5	C6	C7	C8
Mean ± SD	5.08 (±1.85)	6.35 (±1.82)	5.50 (±1.80)	4.46 (±1.51)	5.62 (±1.89)	5.35 (±2.21)	4.85 (±2.44)	7.65 (±1.57)

**Table 11:** Data included in the boxplot of palatability score results of A1 to A6 and C1 to C8.

	A1	A2	A3	A4	A5	A6	C1	C2	C3	C4	C5	C6	C7	C8
**Mean**	1.6	3.4	3.8	4.91	4.03	4.63	5.08	6.35	5.5	4.46	5.62	5.35	4.85	7.65
**Median**	1	3	3	5	4	5	5	6	5	4	5	5.5	5	8
**Quartiles mean**	1.5	3	3.5	4.5	3.5	4.5	5	6.5	5.5	4.75	5.75	5.25	5	7.75
**QCD**	0.33	0.33	0.43	0.33	0.43	0.33	0.20	0.23	0.27	0.16	0.22	0.33	0.40	0.16

**Table 12:** Results of palatability test. Level (%) of A1 to A6’s taste attributes for participants (*n* = 15).

Formulation Characteristics	A1	A2	A3	A4	A5	A6
Sucralose % *w*/*w*	0.0	0.05	0.05	0.05	0.05	0.05
Presence of flavoring agent (number of)	No	No	Yes (one)	Yes (one)	Yes (one)	Yes (one)
**Taste attributes results**						
**Sourness**	14.29%	31.82%	19.23%	30.77%	37.04%	30.77%
**Bitterness**	66.67%	36.36%	42.31%	30.77%	33.33%	34.62%
**Sweetness**	0.00%	13.64%	15.38%	15.38%	7.41%	11.54%
**Delicate**	0.00%	0.00%	3.85%	0.00%	11.11%	3.85%
**Strong**	19.05%	18.18%	19.23%	23.08%	11.11%	19.23%

**Table 13:** Results of palatability test. Level (%) of C1 to C8’s taste attributes for participants (*n* = 13).

Formulation	C1	C2	C3	C4	C5	C6	C7	C8
Sucralose % *w*/*w*	0.20	0.20	0.20	0.20	0.20	0.20	0.20	0.20
Presence of flavoring agent (number of)	No	Yes	Yes (one)	Yes (one)	Yes (one)	Yes (two)	Yes (two)	Yes (two)
**Taste attributes results**								
**Sourness**	16.67%	26.92%	17.24%	32.00%	32.26%	33.33%	27.59%	35.71%
**Bitterness**	33.33%	30.77%	34.48%	40.00%	29.03%	23.33%	27.59%	17.86%
**Sweetness**	12.50%	7.69%	6.90%	8.00%	12.90%	20.00%	17.24%	14.29%
**Delicate**	12.50%	15.38%	17.24%	12.00%	6.45%	10.00%	13.79%	17.86%
**Strong**	25.00%	19.23%	24.14%	8.00%	19.35%	13.33%	13.79%	14.29%

**Table 14:** Principal components analysis (PCA) for the studied variables.

Variable	PC1	PC2	PC3	PC4	PC5	PC6
Age	−0.019	0.742	−0.048	0.071	−0.664	0.001
Smell pleasantness	0.347	−0.446	0.173	−0.553	−0.580	0.089
Smell identification	0.030	0.413	0.686	−0.459	0.362	0.130
Taste pleasantness	0.672	0.134	−0.009	0.054	0.135	−0.713
Taste identification	0.112	−0.205	0.666	0.674	−0.208	0.070
Final palatability score	0.643	0.140	−0.233	0.148	0.172	0.679

**Table 15:** Stability CARV assay data of C8_P2 at different storage temperatures for 6 months.

C8_P2 Formulation				
Storage Temperature	T0	1 Month	3 Months	6 Months
% CARV Assay (Mean of three batches ± SD)				
25 °C	100.80 ± 0.56	101.40 ± 0.56	99.28 ± 0.77	100.06 ± 0.67
30 °C		101.76 ± 1.42	100.79 ± 0.88	101.81 ± 1.76
40 °C		102.57 ± 1.27	101.34 ± 1.72	102.26 ± 1.33

**Table 16:** In-use Stability CARV assay data of one batch of C8_P2 at different temperatures for 15 days.

C8_P2 Formulation				
Storage Temperature	T0	7 Days	10 Days	15 Days
% CARV Assay				
25 °C	100.80	99.66	99.59	99.40

**Table 17:** In-use Stability CARV results of one batch of C8_P2 at 25 °C for 15 days.

C8_P2 Formulation				
	Appearance	pH	% CARV Assay	Microbiological Testing
T0	clear, translucent solution without any undissolved particles	2.47	100.80	conforming
15-day	No changes	No changes	99.40	conforming

## Data Availability

Data are unavailable due to privacy restrictions.

## Supplementary Materials

The following supporting information can be downloaded at https://www.mdpi.com/article/10.3390/pharmaceutics16010030/s1, File S1 (Questionnaire 1. Tests 1 and 2); File S2 (Questionnaire 2. Test 3); File S3 (Ethic protocol); File S4 (C8_P2 solution-representative image).
