# Supplementary material for: Palatability and Stability Studies to Optimize a Carvedilol Oral Liquid Formulation for Pediatric Use
Source: Pharmaceutics. 2023 Dec 25;16(1):30. doi: 10.3390/pharmaceutics16010030 (PMC10820228; doi:10.3390/pharmaceutics16010030)
Supplement: Supplementary file 1 [file pharmaceutics-16-00030-s001.zip › S1-Questionnaire 1. Test 1 and Test 2.pdf]

TEST DE EVALUACIÓN SENSORIAL

Título del estudio: ESTUDIO DE PALATABILIDAD DE UNA NUEVA FORMULACIÓN DE CARVEDILOL LÍQUIDA ORAL INDICADA PARA PEDIATRÍA
Código de protocolo: PDG_CARVEDILOL_01_01

Información previa:

- A continuación recibirá 10 muestras en total de una formulación destinada a la población pediátrica. En el test 1 recibirá 6 muestras y en el test 2 recibirá 4 muestras.
- Cate toda la cantidad de muestra suministrada. Sólo cátela, no la ingiera.
- Enjuague la boca con agua antes de comenzar el test y cada vez que cambie la muestra.
- Para evitar posibles alteraciones en los resultados, no hable ni comente durante la evaluación.
- Si necesita aclaración sobre el funcionamiento del test, no dude en preguntar.

Panelista nº:

TEST 1: AROMATIZACIÓN

REFERENCIA 1/6_Test 1 nº:

1. Olor

Huela la muestra suministrada.

1.1 El olor percibido:

☐ No me gusta nada ☐ No me gusta mucho ☐ Me gusta ☐ Me gusta mucho

1.2 Con cuál de los siguientes olores identifica la muestra:

☐ Fresa ☐ Manzana ☐ Plátano
☐ Chocolate ☐ Vainilla ☐ Neutro
☐ Ninguno de los anteriores. Especifique:

1.3 Comentarios:

2. Sabor

Cate la cantidad de muestra suministrada, no la ingiera.

2.1 El sabor percibido en el momento de catar la muestra:

☐ No me gusta nada ☐ No me gusta mucho ☐ Me gusta ☐ Me gusta mucho

2.2 Cuáles de los siguientes adjetivos describe mejor el sabor de la muestra (puede marcar más de una opción):

☐ Ácido ☐ Dulce ☐ Intenso
☐ Amargo ☐ Suave ☐ Insípido

2.3 Una vez catada la muestra, ¿nota algún sabor residual?

☐ Sí ☐ No

2.4 En caso afirmativo, lo puede describir como:

☐ No me gusta nada ☐ No me gusta mucho ☐ Me gusta ☐ Me gusta mucho

2.5 Con cuál de los siguientes olores identifica la muestra:

☐ Fresa ☐ Manzana ☐ Plátano
☐ Chocolate ☐ Vainilla ☐ Neutro
☐ Ninguno de los anteriores. Especifique:

2.6 Comentarios

3. ¿Qué puntuación designaría a la fórmula objeto de estudio? (puede indicar decimales)

1 – 2 – 3 – 4 – 5 – 6 – 7 – 8 – 9 – 10

4. Comentarios adicionales

REFERENCIA 2/6_Test 1 nº:

1. Olor

Huela la muestra suministrada.

1.1 El olor percibido:

☐ No me gusta nada ☐ No me gusta mucho ☐ Me gusta ☐ Me gusta mucho

1.2 Con cuál de los siguientes olores identifica la muestra:

☐ Fresa ☐ Manzana ☐ Plátano
☐ Chocolate ☐ Vainilla ☐ Neutro
☐ Ninguno de los anteriores. Especifique:

1.3 Comentarios:

2. Sabor

Cate la cantidad de muestra suministrada, no la ingiera.

2.1 El sabor percibido en el momento de catar la muestra:

☐ No me gusta nada ☐ No me gusta mucho ☐ Me gusta ☐ Me gusta mucho

2.2 Cuáles de los siguientes adjetivos describe mejor el sabor de la muestra (puede marcar más de una opción):

☐ Ácido ☐ Dulce ☐ Intenso
☐ Amargo ☐ Suave ☐ Insípido

2.3 Una vez catada la muestra, ¿nota algún sabor residual?

☐ Sí ☐ No

2.4 En caso afirmativo, lo puede describir como:

☐ No me gusta nada ☐ No me gusta mucho ☐ Me gusta ☐ Me gusta mucho

2.5 Con cuál de los siguientes olores identifica la muestra:

☐ Fresa ☐ Manzana ☐ Plátano
☐ Chocolate ☐ Vainilla ☐ Neutro
☐ Ninguno de los anteriores. Especifique:

2.6 Comentarios

3. ¿Qué puntuación designaría a la fórmula objeto de estudio? (puede indicar decimales)

1 – 2 – 3 – 4 – 5 – 6 – 7 – 8 – 9 – 10

4. Comentarios adicionales

REFERENCIA 3/6_Test 1 nº:

1. Olor

Huela la muestra suministrada.

1.1 El olor percibido:

☐ No me gusta nada ☐ No me gusta mucho ☐ Me gusta ☐ Me gusta mucho

1.2 Con cuál de los siguientes olores identifica la muestra:

☐ Fresa ☐ Manzana ☐ Plátano
☐ Chocolate ☐ Vainilla ☐ Neutro
☐ Ninguno de los anteriores. Especifique:

1.3 Comentarios:

2. Sabor

Cate la cantidad de muestra suministrada, no la ingiera.

2.1 El sabor percibido en el momento de catar la muestra:

☐ No me gusta nada ☐ No me gusta mucho ☐ Me gusta ☐ Me gusta mucho

2.2 Cuáles de los siguientes adjetivos describe mejor el sabor de la muestra (puede marcar más de una opción):

☐ Ácido ☐ Dulce ☐ Intenso
☐ Amargo ☐ Suave ☐ Insípido

2.3 Una vez catada la muestra, ¿nota algún sabor residual?

☐ Sí ☐ No

2.4 En caso afirmativo, lo puede describir como:

☐ No me gusta nada ☐ No me gusta mucho ☐ Me gusta ☐ Me gusta mucho

2.5 Con cuál de los siguientes olores identifica la muestra:

☐ Fresa ☐ Manzana ☐ Plátano
☐ Chocolate ☐ Vainilla ☐ Neutro
☐ Ninguno de los anteriores. Especifique:

2.6 Comentarios

3. ¿Qué puntuación designaría a la fórmula objeto de estudio? (puede indicar decimales)

1 – 2 – 3 – 4 – 5 – 6 – 7 – 8 – 9 – 10

4. Comentarios adicionales

REFERENCIA 4/6_Test 1 nº:

1. **Olor**

Huela la muestra suministrada.

1.1 El olor percibido:

☐ No me gusta nada ☐ No me gusta mucho ☐ Me gusta ☐ Me gusta mucho

1.2 Con cuál de los siguientes olores identifica la muestra:

☐ Fresa ☐ Manzana ☐ Plátano
☐ Chocolate ☐ Vainilla ☐ Neutro
☐ Ninguno de los anteriores. Especifique:

1.3 Comentarios:

2. **Sabor**

Cate la cantidad de muestra suministrada, no la ingiera.

2.1 El sabor percibido en el momento de catar la muestra:

☐ No me gusta nada ☐ No me gusta mucho ☐ Me gusta ☐ Me gusta mucho

2.2 Cuáles de los siguientes adjetivos describe mejor el sabor de la muestra (puede marcar más de una opción):

☐ Ácido ☐ Dulce ☐ Intenso
☐ Amargo ☐ Suave ☐ Insípido

2.3 Una vez catada la muestra, ¿nota algún sabor residual?

☐ Sí ☐ No

2.4 En caso afirmativo, lo puede describir como:

☐ No me gusta nada ☐ No me gusta mucho ☐ Me gusta ☐ Me gusta mucho

2.5 Con cuál de los siguientes olores identifica la muestra:

☐ Fresa ☐ Manzana ☐ Plátano
☐ Chocolate ☐ Vainilla ☐ Neutro
☐ Ninguno de los anteriores. Especifique:

2.6 Comentarios

3. **¿Qué puntuación designaría a la fórmula objeto de estudio? (puede indicar decimales)**

1 – 2 – 3 – 4 – 5 – 6 – 7 – 8 – 9 – 10

4. **Comentarios adicionales**

REFERENCIA 5/6_Test 1 nº:

1. Olor

Huela la muestra suministrada.

1.1 El olor percibido:

☐ No me gusta nada ☐ No me gusta mucho ☐ Me gusta ☐ Me gusta mucho

1.2 Con cuál de los siguientes olores identifica la muestra:

☐ Fresa ☐ Manzana ☐ Plátano
☐ Chocolate ☐ Vainilla ☐ Neutro
☐ Ninguno de los anteriores. Especifique:

1.3 Comentarios:

2. Sabor

Cate la cantidad de muestra suministrada, no la ingiera.

2.1 El sabor percibido en el momento de catar la muestra:

☐ No me gusta nada ☐ No me gusta mucho ☐ Me gusta ☐ Me gusta mucho

2.2 Cuáles de los siguientes adjetivos describe mejor el sabor de la muestra (puede marcar más de una opción):

☐ Ácido ☐ Dulce ☐ Intenso
☐ Amargo ☐ Suave ☐ Insípido

2.3 Una vez catada la muestra, ¿nota algún sabor residual?

☐ Sí ☐ No

2.4 En caso afirmativo, lo puede describir como:

☐ No me gusta nada ☐ No me gusta mucho ☐ Me gusta ☐ Me gusta mucho

2.5 Con cuál de los siguientes olores identifica la muestra:

☐ Fresa ☐ Manzana ☐ Plátano
☐ Chocolate ☐ Vainilla ☐ Neutro
☐ Ninguno de los anteriores. Especifique:

2.6 Comentarios

3. ¿Qué puntuación designaría a la fórmula objeto de estudio? (puede indicar decimales)

1– 2 – 3 – 4 – 5 – 6 – 7 – 8 – 9 – 10

4. Comentarios adicionales

REFERENCIA 6/6_Test 1 nº:

1. **Olor**

Huela la muestra suministrada.

1.1 El olor percibido:

☐ No me gusta nada ☐ No me gusta mucho ☐ Me gusta ☐ Me gusta mucho

1.2 Con cuál de los siguientes olores identifica la muestra:

☐ Fresa ☐ Manzana ☐ Plátano
☐ Chocolate ☐ Vainilla ☐ Neutro
☐ Ninguno de los anteriores. Especifique:

1.3 Comentarios:

2. **Sabor**

Cate la cantidad de muestra suministrada, no la ingiera.

2.1 El sabor percibido en el momento de catar la muestra:

☐ No me gusta nada ☐ No me gusta mucho ☐ Me gusta ☐ Me gusta mucho

2.2 Cuáles de los siguientes adjetivos describe mejor el sabor de la muestra (puede marcar más de una opción):

☐ Ácido ☐ Dulce ☐ Intenso
☐ Amargo ☐ Suave ☐ Insípido

2.3 Una vez catada la muestra, ¿nota algún sabor residual?

☐ Sí ☐ No

2.4 En caso afirmativo, lo puede describir como:

☐ No me gusta nada ☐ No me gusta mucho ☐ Me gusta ☐ Me gusta mucho

2.5 Con cuál de los siguientes olores identifica la muestra:

☐ Fresa ☐ Manzana ☐ Plátano
☐ Chocolate ☐ Vainilla ☐ Neutro
☐ Ninguno de los anteriores. Especifique:

2.6 Comentarios

3. **¿Qué puntuación designaría a la fórmula objeto de estudio? (puede indicar decimales)**

1 – 2 – 3 – 4 – 5 – 6 – 7 – 8 – 9 – 10

4. **Comentarios adicionales**

TEST 2: NIVEL DE DULZOR

A continuación recibirá 4 muestras para que evalúe principalmente el nivel de dulzor de cada una.
Cate la cantidad de muestra suministrada, no la ingiera.

REFERENCIA 1/4_Test 2 nº:

1. Sabor

1.1 El sabor percibido en el momento de catar la muestra:

☐ No me gusta nada ☐ No me gusta mucho ☐ Me gusta ☐ Me gusta mucho

1.2 ¿Qué nivel de dulzor designaría a la fórmula objeto de estudio?

0	1	2	3	4	5	6	7	8	9	10
No reconocible		Suave			Óptimo		Moderado			Fuerte

1.3 Una vez catada la muestra, ¿nota algún sabor residual?

☐ Sí ☐ No

1.4 En caso afirmativo, lo puede describir cómo:

☐ Especifique:

2. Comentarios adicionales

REFERENCIA 2/4_Test 2 nº:

1. Sabor

1.1 El sabor percibido en el momento de catar la muestra:

☐ No me gusta nada ☐ No me gusta mucho ☐ Me gusta ☐ Me gusta mucho

1.2 ¿Qué nivel de dulzor designaría a la fórmula objeto de estudio?

0	1	2	3	4	5	6	7	8	9	10
No reconocible		Suave			Óptimo		Moderado			Fuerte

1.3 Una vez catada la muestra, ¿nota algún sabor residual?

☐ Sí ☐ No

1.4 En caso afirmativo, lo puede describir como:

☐ Especifique:

2. Comentarios adicionales

REFERENCIA 3/4_Test 2 nº:

1. Sabor

1.1 El sabor percibido en el momento de catar la muestra:

☐ No me gusta nada ☐ No me gusta mucho ☐ Me gusta ☐ Me gusta mucho

1.2 ¿Qué nivel de dulzor designaría a la fórmula objeto de estudio?

0	1	2	3	4	5	6	7	8	9	10
No reconocible		Suave			Óptimo		Moderado			Fuerte

1.3 Una vez catada la muestra, ¿nota algún sabor residual?

☐ Sí ☐ No

1.4 En caso afirmativo, lo puede describir cómo:

☐ Especifique:

2. Comentarios adicionales

REFERENCIA 4/4_Test 2 nº:

1. Sabor

1.1 El sabor percibido en el momento de catar la muestra:

☐ No me gusta nada ☐ No me gusta mucho ☐ Me gusta ☐ Me gusta mucho

1.2 ¿Qué nivel de dulzor designaría a la fórmula objeto de estudio?

0	1	2	3	4	5	6	7	8	9	10
No reconocible		Suave			Óptimo		Moderado			Fuerte

1.3 Una vez catada la muestra, ¿nota algún sabor residual?

☐ Sí ☐ No

1.4 En caso afirmativo, lo puede describir cómo:

☐ Especifique:

2. Comentarios adicionales
