# Supplementary material for: Palatability and Stability Studies to Optimize a Carvedilol Oral Liquid Formulation for Pediatric Use
Source: Pharmaceutics. 2023 Dec 25;16(1):30. doi: 10.3390/pharmaceutics16010030 (PMC10820228; doi:10.3390/pharmaceutics16010030)
Supplement: Supplementary file 1 [file pharmaceutics-16-00030-s001.zip › S3.zip!/Dictamen 30.06.23.pdf]

INFORME DEL COMITÉ DE ÉTICA DE LA INVESTIGACIÓN SOBRE ENMIENDAS A PROYECTOS DE INVESTIGACIÓN BIOMÉDICA

El Comité de Ética de la Investigación del Hospital Universitari de Bellvitge, en su reunión de fecha 13 de Julio de 2023 (Acta 18//23), tras examinar toda la documentación presentada sobre el proyecto de investigación con nuestra Ref. **PR103/22**, titulado:

"ESTUDIO DE PALATABILIDAD DE UNA NUEVA FORMULACIÓN DE CARVEDILOL LÍQUIDA ORAL INDICADA PARA PEDIATRÍA", código: **PDG_CARVEDILOL_01_01**

Documentos modificados:

- Protocolo versión de fecha 30/06/23.
- Hoja de información al paciente y consentimiento informado versión de fecha 30/06/23.
- Modificación del código de protocolo, anterior: PDG_CARVEDILOL_01_03.

Presentado por la Sra. Blanca Chiclana Rodríguez, Estudiante de Doctorado, del Departamento de Farmacia, Tecnología Farmacéutica y Fisicoquímica del Universitat de Barcelona, como Doctoranda, y Dirigida por la Dra. Pilar Pérez Lozano y Dra. Encarna García Montoya, del Departamento de Farmacia, Tecnología Farmacéutica y Fisicoquímica de la Universitat de Barcelona, ha acordado emitir INFORME FAVORABLE a la documentación mencionada.

Que la composición actual del Comité de Ética de la Investigación es la siguiente:

Presidente	Dr. Francesc Esteve Urbano	Médico - Medicina Intensiva
Vicepresidenta	Dra. Pilar Hereu Boher	Médico - Farmacología Clínica
Secretario	Dr. Enric Sospedra Martínez	Farmacéutico - Farmacia Hospitalaria
Vocales:	Dr. Jordi Adamuz Tomás	Enfermero – Enfermería
	Sra. Anna Boix Traserra	Derecho - DPD
	Dra. Nuria Calaf Figueras	Farmacéutica – Atención Primaria
	Dra. Concepción Cañete Ramos	Médico - Neumología
	Dra. Sara Cobo Sacristán	Farmacéutica – Farmacia Hospitalaria
	Dr. José Luis Ferreiro Gutiérrez	Médico - Cardiología
	Dra. Ana María Ferrer Artola	Farmacéutica - Miembro sanitario
	Sra. Esther Ferrer Canals	Representante de los pacientes
	Dr. Xavier Fulladosa Oliveras	Médico - Nefrología
	Dr. Carles Lladó i Carbonell	Médico - Urología
	Dra. Sara Larriba Bartolomé	Farmacia - Sanitario
	Sra. Sonia López Ortega	Graduado Social - Atención a la Ciudadanía
	Dr. Antoni Lorente Martínez	Ingeniería – Miembro no sanitario
	Dr. Juan Jesús Martín Liberal	Médico - Oncología Médica
	Dr. Sergio Morchón Ramos	Médico - Medicina Preventiva
	Dr. Miguel Ángel Pavón Ribas	Biólogo- Miembro no sanitario

Dr. Joan Josep Queralt Jiménez	Jurista
Dra. María Teresa Terán Virtus	Médico - Farmacología Clínica

Que este Comité cumple la legislación española vigente para este tipo de proyectos, así como las normas ICH y las Normas de Buena Práctica Clínica.

Que en dicha reunión del Comité de Ética de la Investigación se cumplió el quórum preceptivo legalmente.

Lo que firmo en L'Hospitalet de Llobregat, a 25 de julio de 2023

SOSPEDRA MARTINEZ ENRIQUE - 36986426B

Signat digitalment per SOSPEDRA MARTINEZ ENRIQUE - 36986426B
Data: 2023.07.25 09:51:15 +02'00'

Dr. Enric Sospedra Martínez
