# Supplementary material for: Palatability and Stability Studies to Optimize a Carvedilol Oral Liquid Formulation for Pediatric Use
Source: Pharmaceutics. 2023 Dec 25;16(1):30. doi: 10.3390/pharmaceutics16010030 (PMC10820228; doi:10.3390/pharmaceutics16010030)
Supplement: Supplementary file 1 [file pharmaceutics-16-00030-s001.zip › S3.zip!/Final protocol PDG_CARVEDILOL_01_03 (30.06.23).pdf]

FIRMAS:

Redactado por Estudiante de Doctorado UB:

Blanca Chiclana Rodríguez

Fecha 30/06/23

Revisado por Directora de Tesis Doctoral:

Prof. Dra. Pilar Pérez Lozano

Fecha 30/06/23

Verificado por Directora de Tesis Doctoral

Prof. Dra. Encarna García Montoya

Fecha 30/06/23

Validado por Tutor de Tesis Doctoral:

Prof. Dr. Josep Maria Suñé Negre

Fecha 30/06/23

ÍNDICE

0. JUSTIFICACIÓN NUEVA EDICIÓN......pág 3
1. INTRODUCCIÓN......pág 3
   1.1 Antecedentes......pág 3
   1.2 Justificación del estudio......pág 3
2. OBJETIVOS......pág 3
3. DISEÑO DEL ESTUDIO......pág 4
4. CRITERIOS DE SELECCIÓN DE PARTICIPANTES......pág 5
5. TRATAMIENTO......pág 5
6. MÉTODOS ESTADÍSTICOS Y DETERMINACIÓN DEL TAMAÑO DE MUESTRA: PLAN DE ANÁLISIS ESTADÍSTICO......pág 6
   6.1 Pruebas de aceptabilidad......pág 6
   6.2 Pruebas descriptivas......pág 6
7. ASPECTOS ÉTICOS Y DE CONFIDENCIALIDAD......pág 7
   7.1 Realización ética del estudio......pág 7
   7.2 Consentimiento e información al participante......pág 7
   7.3 Protección de datos personales......pág 8
8. REFERENCIAS......pág 8
9. ANEXOS......pág 9

0. JUSTIFICACIÓN NUEVA EDICIÓN

Se realiza una nueva versión del protocolo para añadir 3 nuevos tratamientos en el estudio. El tratamiento inicial propuesto constaba de las formulaciones descritas en el Anexo IV. Al no obtener resultados conformes, se proponen los tratamientos descritos en el Anexo V.

1. INTRODUCCIÓN

La temática de este proyecto es el desarrollo de una formulación líquida dirigida a la población pediátrica, cuya composición contiene como principio activo carvedilol, la cual es inexistente actualmente en el mercado. Con la finalidad de escoger un saborizante adecuado para dicha formulación es necesario realizar un test de palatabilidad.

1.1 Antecedentes

El carvedilol es un fármaco que posee acción vasodilatadora y es usado clínicamente para tratar enfermedades cardiovasculares como fallo cardíaco o hipertensión. Aunque el carvedilol se ha probado solo en adultos, varios estudios han demostrado su eficacia en niños con fallo cardíaco [1].

La única formulación oral disponible comercializada de carvedilol se presenta en una forma farmacéutica sólida, como comprimido. Es necesario, por lo tanto, el desarrollo de una formulación líquida de carvedilol para pediatría. Por un lado, niños menores de 7 años generalmente no son capaces de digerir formas farmacéuticas sólidas; y por otro, a la hora de escoger una dosis determinada para cada paciente por peso de éste, es bastante más sencillo realizar este proceso con una formulación líquida, escogiendo los mililítros necesarios a través de una jeringuilla [2].

1.2 Justificación del estudio de palatabilidad

La palatabilidad es uno de los principales elementos a tener en cuenta para garantizar una correcta aceptabilidad por parte de los pacientes pediátricos, y en consecuencia una buena adherencia al tratamiento. A la hora de escoger un saborizante adecuado para las formulaciones pediátricas, se aconseja que la formulación posea un sabor neutral o un sabor generalmente aceptado por esta población. Los sabores fuertes pueden ser desagradables si la frecuencia de administración de dicha formulación es alta, como por ejemplo en enfermedades crónicas. Lo ideal es que la palatabilidad de una formulación pediátrica sea satisfactoria por sí sola, sin necesidad de mezclar la formulación con bebida (como leche) o comida [3].

Asimismo, en la población infantil se da un crecimiento y un desarrollo rápidos asociados a cambios en diversos órganos, en la composición corporal, en los enlaces proteicos, en los mecanismos de transporte activos y en vías metabólicas, que hay que tener en cuenta. Además de

ser una tarea complicada, es también un paso crítico en el desarrollo de formulaciones pediátricas ya que algunos excipientes aceptables en formulaciones para pacientes adultos no son adecuados para su utilización pediátrica. Por ello, la incorporación de cualquier excipiente en una formulación pediátrica debe estar correctamente justificado, y más especialmente el uso de saborizantes o colorantes [4].
Debido a la dificultad para realizar este estudio en niños, se llevará a cabo en adultos y posteriormente se realizará su correcta extrapolación.

2. OBJETIVOS DEL ESTUDIO

Objetivo principal
Selección de la formulación con mejor propiedades organolépticas. Conocer el saborizante más aceptado por la población de estudio (panelistas): los panelistas evaluarán el sabor de diferentes formulaciones, a través de un cuestionario, un test de evaluación sensorial.

Objetivo secundario
Extrapolación de la preferencia del sabor por parte de población adulta a una preferencia espererada por parte de población pediátrica. Determinar si los adultos pueden predecir la preferencia de palatabilidad de los niños (población a la que va dirigida la formulación de estudio).

3. DISEÑO DEL ESTUDIO

Descripción del estudio: el estudio a realizar consiste en la evaluación sensorial de una formulación líquida base para uso pediátrico que contiene como principio activo carvedilol, formulada con diferentes saborizantes con el objetivo de conocer el saborizante más aceptado por la población de estudio (panelistas). Los panelistas serán miembros (académicos y facultativos) de la Facultad de Farmacia de la Universidad de Barcelona (Departamento de Tecnología Farmacéutica) e investigadores del SDM (Servei de Desenvolupament del Medicament).

- **Investigador principal:** BLANCA CHICLANA RODRÍGUEZ (Estudiante de Doctorado Universitat de Barcelona)
- **Investigadores:** PILAR PÉREZ LOZANO Y ENCARNA GARCÍA MONTOYA (Directoras de Tesis Doctoral), JOSEP MARÍA SUÑÉ NEGRE (Tutor de Tesis Doctoral)
- **Centro de estudio:** Unitat de Tecnologia Farmacèutica. Facultat de Farmàcia i Ciències de l'Alimentació (Universitat de Barcelona).
  Av. Joan XXIII s/n, Edifici A, Escala A, planta baixa
  Barcelona 08028

Tel/fax: 93 403 59 37

- **Tipo de estudio:** simple-ciego, sólo hay enmascaramiento por parte de los panelistas. Controlado con placebo, también se catará una formulación sin saborizante ("placebo"). Uni-centro. Aleatorizado.
- **Número de participantes:** 5-15.
- **Modelo observacional:** controles (adultos sanos)
- **Perspectiva:** prospectivo.
- **Título oficial:** Estudio de palatabilidad de una nueva formulación de carvedilol líquida oral indicada para pediatría
- **Fecha de inicio del estudio:** marzo-julio 2022
- **Duración del estudio:** < 24 h. El estudio solo consta de una sesión de 1 h aproximadamente.

4. CRITERIOS DE SELECCIÓN DE PARTICIPANTES

Criterios de inclusión:

1) Sujetos mayores de 18 años.
2) Sujetos sanos en el momento del estudio.
3) Sujetos capaces de evaluar el test de palatabilidad, según el criterio del investigador.
4) Sujetos que hayan dado previamente su consentimiento informado sobre la participación en el estudio.

Criterios de exclusión:

1) Sujetos menores de 18 años.
2) Mujeres embarazadas o en periodo de lactancia.
3) Sujetos con un proceso infeccioso activo en el momento de la inclusión, tanto crónico como agudo.
4) Sujetos con historia documentada de sensibilidad/idiosincrasia al carvedilol o excipientes de la fórmula (ácido málico y diferentes saborizantes).
5) Sujetos que posean alguna condición que influya en el sentido del gusto o el olfato (como un proceso infeccioso respiratorio, enfermedad febril, mucositis) o que están tomando una medicación que pueda influir.

5. TRATAMIENTO

La composición de las muestras a catar por los panelistas que participarán en el estudio se indica en la Tabla 1. Los panelistas sólo catarán las diferentes muestras, no las ingerirán. Este test de evaluación sensorial se encuentra detallado en el ANEXO I.

Componentes	Función	% p/p en la fórmula
Carvedilol	Principio activo	0,5
Ácido málico	Regulador pH	6
Saborizante	Saborizante, aromatizante	0,15-0,2
Agua desionizada	Vehículo	csp 100 %

Tabla 1: Composición de las muestras que participarán en el estudio

6. MÉTODOS ESTADÍSTICOS Y DETERMINACIÓN DEL TAMAÑO DE MUESTRA: PLAN DE ANÁLISIS ESTADÍSTICO

6.1 Pruebas de aceptabilidad:

En las pruebas de aceptabilidad se usa una escala hedónica para categorizar el nivel de aceptabilidad de un producto o varios. En este tipo de pruebas se asume que el nivel de aceptabilidad del consumidor existe en un continuo, no necesariamente hay el mismo nivel de escala entre me gusta mucho y me gusta, que entre me disgusta mucho y me disgusta. En este estudio se utilizará una escala con cuatro niveles de aceptabilidad: no me gusta nada, no me gusta mucho, me gusta, me gusta mucho. Se expone un ejemplo de pregunta de aceptabilidad a continuación:

1. **Sabor** .

1.1 El sabor percibido en el momento de catar la muestra:

☐ No me gusta nada ☐ No me gusta mucho ☐ Me gusta ☐ Me gusta mucho

Análisis de datos: Se realiza a través de medida de tendencia central y t-test para definit diferencias. En este caso, la escala es de 4 puntos por lo que se le asigna un valor cada nivel: no me gusta nada "1" hasta me gusta mucho "4". Para observar si los productos tienen la aceptación de los panelistas (traducida en escala numérica) determinamos la media y desviación estándar y para verificar la diferencia entre ambos productos calculamos el valor t para una prueba independiente.

6.2 Pruebas descriptivas:

Dentro de las pruebas descriptivas podemos encontrar pruebas del perfil del sabor. Mediante esta técnica se reportan percepciones, no se hacen preguntas acerca de la aceptabilidad del producto. En este caso, se evaluará lo siguiente:

1. **Sabor** .

1.2 Cuáles de los siguientes adjetivos describe mejor el sabor de la muestra (puede marcar más de una opción):

☐ Ácido ☐ Dulce ☐ Intenso
☐ Amargo ☐ Suave ☐ Insípido

1.3 Con cuál de los siguientes sabores identifica la muestra:

☐ Fresa ☐ Plátano ☐ Manzana
☐ Tutti fruti ☐ Chocolate ☐ Vainilla
☐ Neutro ☐ Ninguno de los anteriores. Especifique:

1.4 Una vez catada la muestra, ¿nota algún sabor residual?

☐ Sí ☐ No

Análisis de datos: Para analizar los datos en este tipo de pruebas se realizan análisis descriptivos.

- Estadística paramétrica y medidas de tendencia central y varianza.
- Prueba de significancia estadística de las diferencias entre dos productos: t-test entre dos productos, Prueba de Duncan, Tukey y LSD.
- Análisis de varianza en el caso de comparaciones entre más de dos productos.

Se debe tener en cuenta ciertos criterios dentro del análisis como:

- Cada atributo descriptivo se analiza por separado.
- Los datos se describen en medias, DE, EE para cada producto y cada atributo.
- Se pueden realizar gráficas o tabulaciones de resultados.

7. ASPECTOS ÉTICOS Y DE CONFIDECIALIDAD

7.1 Realización ética del estudio

Este estudio se realizará de acuerdo con la Declaración de Helsinki sobre principios éticos para la investigación médica en sujetos humanos, adoptada por la Asamblea General de la Asociación Médica Mundial (1996). Además, el estudio se realizará de acuerdo con el protocolo y con las directrices de la conferencia internacional sobre armonización (ICH) y los requisitos normativos para las instituciones participantes. El estudio se realizará según un protocolo revisado por un CEIC; el estudio se realizará por personas científica y cualificadas; los beneficios del estudio están en proporción con los riesgos; se respetarán los derechos y el bienestar de los sujetos; los investigadores que realizan el estudio no encuentran que los riesgos superen los posibles beneficios y cada sujeto facilitará su consentimiento informado sin coacción. Al tratarse de un estudio de palatabilidad, la formulación medicamentosa no se tragará sino que sólo se catará, por lo que no debería de haber ningún riesgo asociado para el sujeto de estudio.

7.2 Consentimiento e información al participante

El investigador presentará un modelo de consentimiento informado apropiadamente realizado, por escrito, en cumplimiento con la BPC según las directrices de la ICH y los requisitos legales

locales al CEIC para su revisión y aprobación antes de iniciar el estudio. Antes de introducir sujetos en el estudio, se revisará una copia de consentimiento informado aprobado por el CEIC con el posible participante, y se firmará y fechará. El investigador proporcionará una copia del formulario de consentimiento informado firmado de cada sujeto y conservará una copia en el archivo del estudio del sujeto.
El consentimiento informado se encuentra en el ANEXO II.

7.3 Protección de datos personales

Este estudio no precisa de la utilización de datos identificativos o identificables de los participantes, ni cualquier otra información más allá de la que figura en el Anexo I, Test de evaluación sensorial. En el Anexo I, cada caso se identificará mediante un código numérico otorgado por una persona independiente del equipo investigador. Esta persona será la única que conozca el vínculo entre el código y el participante. La base de datos del estudio (fichero de investigación clínica) se conservará en un directorio propio del Servei de Desenvolupament del Medicament de la Facultat de Farmàcia i Ciències de l'Alimentació.

En las publicación o difusión de los resultados no se incluirá ningún dato de tipo personal, garantizándose en todo momento el anonimato de los participantes en el estudio de palatabilidad, de acuerdo co lo establecido por la normativa legal vigente: Reglamento (UE) 2016/679 del Parlamento Europeo y del Consejo de 27 de abril de 2016 (RGPD) relativo a la protección de las personas físicas en lo que respecta al tratamiento de datos personales y a la libre circulación de estos datos; y Ley Orgánica 3/2018 de 5 de diciembre, de Protección de datos Personales y garantía de derechos digitales.

8. REFERENCIAS

1. Beattie K, Phadke G, Novakovic J. Carvedilol. Profiles Drug Subst Excip Relat Methodol. 2013;38:113-57.
2. Buontempo F, Bernabeu E, Glisoni R and Quiroga E. Carvedilol stability in paediatric oral liquid formulations. Farm Hosp. 2010;34(6):293-297.
3. Rouaz K, Chiclana-Rodríguez B, Nardi-Ricart A, Suñé-Pou M, Mercadé-Frutos D, Suñé-Negre JM, Pérez-Lozano P, García-Montoya E. Excipients in the Paediatric Population: A Review. Pharmaceutics. 2021 Mar 13;13(3):387.
4. European Medicines Agency. ICH E11(R1) Guideline on Clinical Investigation of Medicinal Products in the Pediatric Population. Step 5 [Internet]. Available online: https://www.ema.europa.eu/en/ich-e11r1-step-5-guideline-clinical-investigation-medicinal- products-pediatric-population (accessed on 2 March 2022).

Facultat de Farmàcia i Ciènces de l'Alimentació
Av. Joan XXIII, 27-31.
08028 Barcelona
Tel. / Fax. 93 402 45 46
www.ub.edu/sdm
jmsune@ub.edu

9. ANEXOS

ANEXO I: TEST DE EVALUACIÓN SENSORIAL

Información previa:

- A continuación recibirá X MUESTRAS de una formulación destinada a la población pediátrica.
- Enjuague la boca con agua antes de comenzar el test y cada vez que cambie la muestra.
- Para poder realizar una correcta evaluación del sabor, tome toda la cantidad de muestra administrada.
- Para evitar posibles alteraciones en los resultados, no hable ni comente durante la evaluación.
- Si necesita aclaración sobre el funcionamiento del test, no dude en preguntar.

Panelista:

REFERENCIA: X

1. **Olor**

Huela la muestra suministrada.

1.5 El olor percibido:

☐ No me gusta nada ☐ No me gusta mucho ☐ Me gusta ☐ Me gusta mucho

1.6 Con cuál de los siguientes olores identifica la muestra:

☐ Fresa ☐ Plátano ☐ Manzana
☐ Tutti fruti ☐ Chocolate ☐ Vainilla
☐ Neutro ☐ Ninguno de los anteriores. Especifique:

1.7 Comentarios:

2. **Sabor**

Tome toda la cantidad de muestra suministrada. Sólo cátela.

2.1 El sabor percibido en el momento de catar la muestra:

☐ No me gusta nada ☐ No me gusta mucho ☐ Me gusta ☐ Me gusta mucho

2.2 Cuáles de los siguientes adjetivos describe mejor el sabor de la muestra (puede marcar más de una opción):

☐ Ácido ☐ Dulce ☐ Intenso
☐ Amargo ☐ Suave ☐ Insípido

2.3 Una vez catada la muestra, ¿nota algún sabor residual?

☐ Sí ☐ No

2.4 En caso afirmativo, lo puede describir como:

☐ No me gusta nada ☐ No me gusta mucho ☐ Me gusta ☐ Me gusta mucho

2.5 Con cuál de los siguientes sabores identifica la muestra:

☐ Fresa ☐ Plátano ☐ Manzana

☐ Tutti fruti ☐ Chocolate ☐ Vainilla
☐ Neutro ☐ Ninguno de los anteriores. Especifique:

2.6 Comentarios

3. ¿Qué puntuación designaría a la fórmula objeto de estudio? (puede indicar decimales)

1 – 2 – 3 – 4 – 5 – 6 – 7 – 8 – 9 – 10

4. Comentarios adicionales

ANEXO II: CONSENTIMIENTO INFORMADO

Título del estudio: **ESTUDIO DE PALATABILIDAD DE UNA NUEVA FORMULACIÓN DE CARVEDILOL LÍQUIDA ORAL INDICADA PARA PEDIATRÍA**
Código de protocolo: **PDG_CARVEDILOL_01_01**

Yo, <<nombre y apellidos del participante>>

☐ He leído la hoja de información que se me ha entregado sobre el estudio.

☐ He podido hacer preguntas sobre el estudio.

☐ He recibido suficiente información sobre el estudio.

☐ He hablado con la Investigadora: BLANCA CHICLANA RODRÍGUEZ

☐ Comprendo que mi participación es voluntaria.

☐ Comprendo que puedo retirarme del estudio:

- Cuando quiera.
- Sin tener que dar explicaciones.
- Sin que esto repercuta en mis cuidados médicos.

Recibiré una copia firmada y fechada de esta hoja de información y consentimiento informado
Presto libremente mi conformidad para participar en el estudio, confirmo que he leído el Apéndice 1 y estoy conforme con su contenido.

Firma del participante

Fecha: ____/____/____

Firma del investigador

Fecha: ____/____/____

ANEXO III: HOJA DE INFORMACIÓN AL PARTICIPANTE

TÍTULO DEL ESTUDIO: ESTUDIO DE PALATABILIDAD DE UNA NUEVA FORMULACIÓN DE CARVEDILOL LÍQUIDA ORAL INDICADA PARA PEDIATRÍA
CÓDIGO DEL ESTUDIO: **PDG_CARVEDILOL_01_01**
INVESTIGADOR PRINCIPAL: **BLANCA CHICLANA RODRIGUEZ (Doctoranda UB)**
CENTRO: UNITAT DE TECNOLOGIA FARMACÈUTICA, FACULTAT DE FARMÀCIA I CIÈNCIES DE L'ALIMENTACIÓ. UNIVERSITAT DE BARCELONA

INTRODUCCIÓN

Nos dirigimos a usted para informarle sobre un estudio de palatabilidad en el que se le invita a participar. El estudio ha sido aprobado por el Comité de Ética de la Investigación con medicamentos del Hospital Universitari de Bellvitge.

Nuestra intención es tan sólo que usted reciba la información correcta y suficiente para que pueda evaluar y juzgar si quiere o no participar en este estudio. Para ello lea esta hoja informativa con atención y nosotros le aclararemos las dudas que le puedan surgir después de la explicación. Además, puede consultar con las personas que considere oportuno.

PARTICIPACIÓN VOLUNTARIA Y RETIRADA DEL ESTUDIO

Debe saber que su participación en este estudio es voluntaria y que puede decidir no participar y retirar el consentimiento en cualquier momento, sin que por ello se altere la relación con la investigadora ni con sus compañeros de Departamento. También se le podrá retirar del estudio si en cualquier momento se le detectase algún tipo de intolerancia o malestar relacionados con el tratamiento.

DESCRIPCIÓN GENERAL DEL ESTUDIO

El estudio a realizar consiste en la evaluación sensorial de una formulación líquida base para uso pediátrico que contiene como principio activo carvedilol, formulada con diferentes saborizantes con el objetivo de conocer el saborizante más aceptado por los participantes. Usted tendrá que rellenar un cuestionario anónimo sobre estas formulaciones, y puntuar cada una de ellas. La duración del estudio será tan sólo de una sesión de una hora aproximadamente.

El estudio se realizará en voluntarios de ambos sexos sanos, miembros (académicos y facultativos) de la Facultad de Farmacia de la Universidad de Barcelona (Departamento de Tecnología Farmacéutica) e investigadores del SDM (Servei de Desenvolupament del Medicament) que cumplan con los criterios de selección. El estudio incluirá entre 5-15 participantes.

Es importante resaltar que no se le va a administrar la formulación, dado que únicamente se catarán las diferentes formulaciones durante breves segundos, y no las ingerirán.

COMPENSACIÓN ECONÓMICA

No se prevé ningún tipo de compensación económica durante el estudio.

BENEFICIOS Y RIESGOS DERIVADOS DE SU PARTICIPACIÓN EN EL ESTUDIO

Usted no obtendrá ningún beneficio directo por participar en el estudio. No obstante, la información que se obtenga puede beneficiar en un futuro a pacientes pediátricos u otros pacientes que necesiten tomar esta medicación. Al finalizar la investigación podrá ser informado, si lo desea, sobre los principales resultados y las conclusiones generales del estudio.

No se prevé ningún efecto adverso ya que como se ha indicado anteriormente, no se ingiere la formulación, sólo se cata. Aun así, si hubiera cualquier tipo de reacción adversa como por ejemplo de hipersensibilidad a alguno de los componentes de la formulación, la investigadora estará presente durante el procedimiento y será monitorizado continuamente.

CONFIDENCIALIDAD

El tratamiento de los datos personales de los particpantes en el marcoo del estudio se regirá por lo establecido en el Reglamento (UE) 2016/679 del Parlamento Europeo y del Consejo de 27 de abril de 2016 (RGPD) relativo a la protección de las personas físicas en lo que respecta al tratamiento de datos personales y a la libre circulación de estos datos; y Ley Orgánica 3/2018 de 5 de diciembre, de Protección de datos Personales y garantía de derechos digitales.

La UNIVERSIDAD DE BARCELONA, (con CIF Q0818001J y domicilio en la Gran Via de les Corts Catalanes, 585-08007 Barcelona) como responsable del tratamiento de los datos personales, le informa que puede conctactar con el Delegado de Protección de Datos mediante escrito a la dirección postal (Travessera de les Corts, 131-159, Pavelló Rosa, 08028 – Barcelona), o mediante un mensaje de correo electrónico a protecciodedades@ub.edu.

En este estudio no se precisa ningún dato personal más allá de los datos que figuran en el test de evaluación sensorial que, por otra parte, únicamente contempla datos telacionados con la palatibilidad de las formulaciones testadas. En todo caso, cada cuestionario se identificará mediante un códiigo numérico otorgado por una persona del Departamento, independiente de equipo, que será la única que conocerá el vínculo entre dicho código y la identicación del participante.

Usted tiene derecho a acceder a sus datos, a solicitar la rectificación de los datos inexactos y a solicitar su supresión, así como a limitar el tratamiento, a oponerse y a retirar el consentimiento de su uso para determinadas finalidades. Estos derechos los puede ejercer mediante escrito a la dirección postal o mediante un mensaje de correo electrónico a la dirección mencionada en el párrafo anterior. Así mismo, le informamos de su derecho a presentar una reclamación ante la Agencia Catalana de Protección de Datos en el caso de cualquier actuación de la Universitat de Barcelona que considere que vulnera sus derechos.

Los datos codificados no se compartirán con fines de comercialización directa ni para otros fines que no sean obligaciones legales o que no se consideren investigación científica de conformidad con la legislación vigente en materia de protección de datos.

ANEXO IV: FORMULACIONES – TRATAMIENTO DEL ESTUDIO

La composición de las fórmulas a catar por los panelistas que participarán en el estudio se indica en la Tabla 1. En la Tabla 2 se muestra la función de cada componente de la fórmula. Los panelistas sólo catarán las diferentes muestras, no las ingerirán.

Fórmula	% p/p carvedilol	% p/p ácido málico	Saborizante				
			Tipo saborizante	% p/p saborizante	Proveedor	Lote	Fecha caducidad
F8	0,5	6	Chocolate (white)	0,15	KERRY	8900161472	08/11/2022
F9	0,5	6	Strawberry	0,15	KERRY	8900161471	17/06/2023
F10	0,5	6	Strawberry	0,2	GIVAUDAN	D9A0179922	08/10/2023
F11	0,5	6	Banana	0,2	GIVAUDAN	D9A0176987	01/09/2022
F12	0,5	6	Apple Pear	0,2	GIVAUDAN	D9A0179749	08/10/2022
F13	0,5	6	Tropical Fruit	0,2	GIVAUDAN	D9A0175019	11/08/2022
F14	0,5	6	Vanilla	0,2	GIVAUDAN	D9A0181805	28/04/2023
F15	0,5	6	French Vanilla	0,2	GIVAUDAN	D9A0184003	30/05/2023
F16	0,1	0,8	SIN AROMA	0	-	-	-
F17	0,5	6	SIN AROMA	0	-	-	-

Tabla 1: Composición de las formulaciones que participarán en el estudio

Componentes	Función	% p/p en la fórmula
Carvedilol	Principio activo	0,1 - 0,5
Ácido málico	Regulador pH	0,8 - 6
Saborizante	Saborizante, aromatizante	0,15 - 0,2
Agua desionizada	Vehículo	csp 100 %

Tabla 2: Composición general y función de cada componente de las formulaciones dele estudio

ANEXO V: NUEVOS TRATAMIENTOS DEL ESTUDIO

Las formulaciones descritas en el Anexo IV no obtuvieron resultados conformes en el estudio de palatabilidad por parte de los panelistas. Se proponen por lo tanto 3 nuevos tratamientos.

TRATAMIENTO PROPUESTO 1: Elección concentración edulcorante

Los panelistas catarán diferentes formulaciones, las cuales no contienen ningún tipo de saborizante, sólo contienen sucralosa en diferentes concentraciones. El objetivo de esta prueba es conocer la concentración de sucralosa más aceptada por los panelistas. Estas formulaciones, B1-B4, se describen en la Tabla 1.

Fórmula	% p/p carvedilol	% p/p ácido málico	% p/p sucralosa
B1	0,10	0,80	0,02
B2	0,10	0,80	0,05
B3	0,10	0,80	0,10
B4	0,10	0,80	0,20

Tabla 1: *Composición de las formulaciones que participarán en el estudio. Elección concentración de sucralosa*

TRATAMIENTO PROPUESTO 2: Elección de saborizante

La finalidad de este estudio es escoger el saborizante más aceptado por la población de estudio, con una mínima concentración de edulcorante. La composición de estas formulaciones, indicadas como formulación A1 – A6, se describe en la Tabla 2.

Fórmula	% p/p carvedilol	% p/p ácido málico	% p/p sucralosa	Saborizante				
				Tipo saborizante	% p/p	Proveedor	Lote	Fecha de reanálisis
A1	0,10	0,80	-	-	-	-	-	-
A2	0,10	0,80	0,05	-	-	-	-	-
A3	0,10	0,80	0,05	Chocolate white (LAB19462)	0,20	KERRY	8900161472	08/11/2022
A4	0,10	0,80	0,05	Strawberry (F-10815)	0,20	KERRY	8900161471	17/06/2023
A5	0,10	0,80	0,05	Strawberry (S-139770)	0,20	GIVAUDAN	D9A019922	08/10/2023
A6	0,10	0,80	0,05	Apple Pear (S-144937)	0,20	GIVAUDAN	D90179749	08/10/2022

Tabla 2: Composición de las formulaciones que participarán en el estudio. Elección de saborizante

Facultat de Farmàcia i Ciències de l'Alimentació
Av. Joan XXIII, 27-31.
08028 Barcelona
Tel. / Fax. 93 402 45 46
www.ub.edu/sdm
jmsune@ub.edu

TRATAMIENTO PROPUESTO 3: Elección combinación sucralosa + saborizante:

El objetivo de esta prueba es encontrar la formulación con una combinación de sucralosa 0,20 % y diferente/s saborizante/s más aceptada y con resultados conformes por la población de estudio. Se proponen las formulaciones C1 – C8 descritas en la Tabla 3 de este anexo.

Fórmula	% p/p carvedilol	% p/p ácido málico	% p/p sucralosa	Saborizantes					
				Tipo saborizante		% p/p	Proveedor	Lote	Fecha de reanálisis
C1	0,10	0,80	0,20	-		-	-	-	-
C2	0,10	0,80	0,20	Lemon (S-080551)		0,10	Givaudan	D9G0000063	12/03/2024
C3	0,10	0,80	0,20	Zumo de naranja (75935-71)		0,10	Givaudan	D9A0208621	08/12/2023
C4	0,10	0,80	0,20	Limón (L-249791)		0,20	Givaudan	D9A0202620	06/06/2023
C5	0,10	0,80	0,20	Apple Pear (S-144937)		0,20	Givaudan	D9A0208302	01/12/2023
C6	0,10	0,80	0,20	Saborizante 1	Limón (L-249791)	0,15	Givaudan	D9A0202620	06/06/2023
				Saborizante 2	Zumo de naranja (75935-71)	0,05	Givaudan	D9A0208621	08/12/2023
C7	0,10	0,80	0,20	Saborizante 1	Limón (L-249791)	0,15	Givaudan	D9A0202620	06/06/2023
				Saborizante 2	Apple Pear (S-144937)	0,05	Givaudan	D9A0208302	01/12/2023
C8	0,10	0,80	0,20	Saborizante 1	Limón (L-249791)	0,15	Givaudan	D9A0202620	06/06/2023
				Saborizante 2	Cola (CS-492-239-7)	0,05	Givaudan	D9A0198089	27/06/2024

Tabla 3: Composición de las formulaciones que participaron en el estudio. Elección final combinación sucralosa + saborizante
